# CDPK1 from Ginger Promotes Salinity and Drought Stress Tolerance without Yield Penalty by Improving Growth and Photosynthesis in *Nicotiana tabacum*

**DOI:** 10.1371/journal.pone.0076392

**Published:** 2013-10-23

**Authors:** Padmanabhan Jayanthi Vivek, Narendra Tuteja, Eppurathu Vasudevan Soniya

**Affiliations:** 1 Plant Molecular Biology Division, Rajiv Gandhi Centre for Biotechnology, Thiruvananthapuram, Kerala, India; 2 Plant Molecular Biology Group, International Centre for Genetic Engineering and Biotechnology, New Delhi, India; RIKEN Center for Sustainable Resource Science, Japan

## Abstract

In plants, transient changes in calcium concentrations of cytosol have been observed during stress conditions like high salt, drought, extreme temperature and mechanical disturbances. Calcium-dependent protein kinases (CDPKs) play important roles in relaying these calcium signatures into downstream effects. In this study, a stress-responsive CDPK gene, ZoCDPK1 was isolated from a stress cDNA generated from ginger using rapid amplification of cDNA ends (RLM-RACE) – PCR technique and characterized its role in stress tolerance. An important aspect seen during the analysis of the deduced protein is a rare coupling between the presence of a nuclear localization sequence in the junction domain and consensus sequence in the EF-hand loops of calmodulin-like domain. *ZoCDPK1* is abundantly expressed in rhizome and is rapidly induced by high-salt stress, drought, and jasmonic acid treatment but not by low temperature stress or abscissic acid treatment. The sub-cellular localization of ZoCDPK1-GFP fusion protein was studied in transgenic tobacco epidermal cells using confocal laser scanning microscopy. Over-expression of ginger CDPK1 gene in tobacco conferred tolerance to salinity and drought stress as reflected by the high percentage of seed germination, higher relative water content, expression of stress responsive genes, higher leaf chlorophyll content, increased photosynthetic efficiency and other photosynthetic parameters. In addition, transgenic tobacco subjected to salinity/drought stress exhibited 50% more growth during stress conditions as compared to wild type plant during normal conditions. T3 transgenic plants are able to grow to maturity, flowers early and set viable seeds under continuous salinity or drought stress without yield penalty. The *ZoCDPK1* up-regulated the expression levels of stress-related genes RD21A and ERD1 in tobacco plants. These results suggest that ZoCDPK1 functions in the positive regulation of the signaling pathways that are involved in the response to salinity and drought stress in ginger and it is likely operating in a DRE/CRT independent manner.

## Introduction

Environmental stresses such as drought, salinity, extreme temperatures, chemical toxicity and oxidative stress are serious threats to agriculture and the natural status of the environment [1]. Most of the physiological processes in plants, including responses to abiotic stresses and hormones involve a transient elevation in cytosolic calcium concentrations [2]. Different calcium sensors recognize specific calcium signatures and transduce them into downstream effects, including altered protein phosphorylation and gene expression patterns [3]. Specific responses to different stimuli could be achieved through variations in the amplitude, duration, location, and frequency of this Ca^2+^-spikes. Calcium-dependent (calmodulin-indepedent) protein kinases (CDPKs) are unique sensor-responder proteins in higher plants that decodes and translates the elevation of Ca^2+^ concentration into enhanced protein kinase activity and subsequent downstream signaling events [4]. 

CDPKs are Ser/Thr protein kinases with molecular mass of 40 to 90 KDa [5]. The basic structural features of CDPKs are conserved. They have a five domain structure with an amino-terminal variable domain, a kinase domain, a junction domain (JD), a regulatory domain (CaM-LD, calmodulin-like domain) and a carboxy terminal variable domain [6]. The N-terminal domains of CDPKs vary in length from 40 to 180 amino acids, and there is no significant homology in the sequence between family members itself [6]. The JD between the kinase and CaM-LD functions as a pseudo-substrate autoinhibitor that inhibits phosphorylation in the absence of Ca^2+^ and keeps the CDPK in a state of low activity [7]. The CaM-LD of CDPKs consist of two structural domains (termed the N and C “lobes”), each containing two EF hand helix-loop-helix Ca^2+^-binding motifs. Activation of CDPK occurs when Ca^2+^ levels rise to fill the two weaker affinity-binding sites in the N-lobe, thereby triggering a conformational change that leads to release of autoinhibitory region [8].

Information available from genomic sequencing, as well as several extensive expressed sequences tag projects, indicates the presence of multigene families of CDPKs in various plants [9,10]. Thirty-four CDPK genes were found in *Arabidopsis* and 31 CDPK genes were found in rice [10-12]. Sequencing projects in other plants including soybean, tomato, and maize also indicated the presence of multigene families of CDPKs [6]. In plants, CDPKs are present as both soluble and membrane-anchored isoforms. CDPKs exhibit multiple locations including the cytosol, nucleus, plasma membrane, endoplasmic reticulum, peroxisomes, mitochondrial outer membrane and oil bodies [13]. In *Arabidopsis thaliana* 9 of 34 isoforms have been localized either in the nucleus, cytoplasm or associated with plasma membrane [14,15]. CDPKs are shown to be involved in regulating gene expression linked with carbon and nitrogen metabolism, phospholipid synthesis, defense responses, stress signaling, ion and water transport, stomatal response, cytoskeletal organization, fertilization and proteasome regulation [13,16,17]. Recently involvement of CDPK in flower morphogenesis is also reported [18]. Dehydration, chilling temperature, salinity and hormones can all induce specific changes in the expression of CDPK genes in *Arabidopsis*, rice, tobacco and wheat [19-21]. Transcription of *AtCPK10* and *AtCPK11* can be rapidly induced by drought and high-salt stresses [22]. Xu et al. [23] demonstrated that CPK6 functions as a positive regulator in *Arabidopsis* responses to salt/drought stress, whereas CPK3 kinase activity was found to be induced by salt and other stresses after transient over-expression in *Arabidopsis* [24]. Transgenic rice constitutively expressing *OsCDPK7* and *OsCDPK13* displayed significantly improved tolerance to cold, salt and drought stress [25,26]. Since phytohormones are known to be involved in abiotic stress responses, CDPK genes have also been found to be regulated after treatment with various plant hormones. CDPK expression was found to be regulated after exposure to jasmonic acid (JA), abscissic acid (ABA), and auxin [27-29]. Recently, maize CDPK11 (ZmCDPK11) is reported to be a component of JA signaling and regulated in response to wounding and touch [30]. Although the participation of CDPKs in different signaling pathways is relatively well documented, still little is known about their role in the cross-talk between these pathways, though the CDPKs cross-talk with MAP kinase signaling is described [31]. In spite of CDPK’s potential importance, the physiological functions of a specific CDPK pathway in most of the plants including ginger are still not clear.

Ginger (*Zingiber officinale* Rosc, Family: Zingiberaceae) is an important spice crop of the world. This valuable cash crop is an herbaceous perennial, the rhizomes of which are used as a spice. It requires a well distributed rainfall during growing season and rhizome growth is better on slightly acidic soil and hence salinity/drought stress early during the growth phase is a menace. Therefore, improvement of drought and salinity tolerance in ginger will greatly reduce ginger management cost, benefiting the environment and ginger industry. Despite numerous studies involving the physiological aspects of this large family, a molecular understanding of the stress signaling mechanisms in *Zingiberaceae* remains unknown. Here we report the isolation and characterization of a multiple stress inducible CDPK from ginger, which apart from having all the signature features of a typical CDPK also have a bipartite nuclear localization sequence (NLS) in its junction domain. Over-expression of *ZoCDPK1* in tobacco plant confers salinity stress tolerance without affecting yield, suggesting a previously un-described pathway in ginger for manipulating stress tolerance in this valuable spice.

## Materials and Methods

### Plant material and stress treatments

*Zingiber officinale* var. Rio-de-Janeiro was used for the present study. Ginger rhizomes were collected from the Kerala Agricultural University, Thiruvananthapuram and maintained in the greenhouse of Rajiv Gandhi Centre for Biotechnology, Thiruvananthapuram at 28°C. The different stress treatments were imposed on four week old plants grown from healthy rhizomes and third fully expanded leaves were harvested after stress treatments. Salt stress was given by immersing the seedling in 400 mM NaCl solution and leaves were harvested at regular intervals of 0, 3, 6, 9, 12, 15 and 24 h post treatment. For drought treatment, the seedlings saturated with deionized water were exposed to the air at room temperature and leaves were harvested at regular intervals of 0, 3, 6, 9, 12, 15 and 24 h after dehydration. Abscissic acid treatment was given on young leaves of intact plants by spraying 100 μM solution and leaves were harvested at regular intervals of 0, 3, 6, 9, 12, 15 and 24 h post treatment. For cold treatment four-week old plants were placed at 4°C and leaves were harvested at 0, 3, 6, 9, 12, 15 and 24 h post treatment. Jasmonic acid was sprayed to young leaves of greenhouse plants at a concentration of 100 μM and leaves were collected at regular intervals of 0, 24, 48, 72 and 96 h after treatment. The collected leaf samples were immediately frozen in liquid nitrogen and stored at -80°C until use. 

### RNA isolation and reverse transcription

All samples including leaves, shoots, rhizomes and leaves after various stress treatments were powdered using liquid nitrogen with mortar and pestle. From the leaf samples total RNA was isolated using TRIzol method (Invitrogen, USA) according to manufacturer’s protocol. Total RNA from rhizome and petiole were isolated using RNeasy^®^ Plant Mini kit (QIAGEN Sciences, USA) as per manufacturer’s instructions. Two micrograms of DNase treated RNA were reverse transcribed using High Capacity cDNA Reverse Transcription kit (Applied Biosystems, USA) following the manufacturer’s instructions. The cDNAs were then stored at -20°C.

### CDPK core fragment production

For core fragment production, salt stress was given on intact plants by placing them in 400 mM NaCl solution and kept under 250 lux light intensity for 16 h and RNA was isolated using TRIzol reagent as described earlier. Two micrograms of DNase treated RNA was reverse transcribed for 60 min at 42°C in a 30 µL reaction volume containing 100 U of M-MLV reverse transcriptase (Promega, USA), 200 μM of each dNTPs, 0.5 µg oligo (dT)_15_ primer, 8 U of RNase inhibitor and 5x buffer for RT. Oligonucleotide primers, Ik1 (5’-GGIGTIATGCA (T/C)(C/A)GIGA(T/C)(T/C)TIAA(A/G)AA-3’) and Ik2 (5’-GTIAT(A/G)AAICCIGAICC(A/G)TCT T(A/G)TC–3’), corresponding to the amino acid sequences GVMHRDLKPEN (sub-domain VIb) and DKDGSGYIT (third EF hand), respectively, which are conserved in CDPKs, were used. A primary PCR was carried out in a 50 µL reaction mixture containing 1.0 μL of cDNA, 5x Buffer with MgCl_2,_ 200 μM of each dNTPs, 20 pmol of each primer, and 3 U of *Taq* DNA polymerase (Promega, USA). The cDNA was denatured at 94°C for 3 min followed by 35 cycles (94°C for 30 sec, 43°C for 30 sec and 72°C for 1 min) and a final incubation at 72°C for 7 min. The amplified product was used for the secondary PCR reaction. A second set of primers Tb1 (5’-CCITAYTAYRTIGCICCIGARGT-3’) and Tb2 (5’-CCYTTYTKCATCATIGCIA CRAAYTC-3’) which bind within the conserved regions of the kinase domain and calcium binding domain were used for a second round PCR using the same above conditions. The PCR product was analyzed through agarose gel (1.5%) electrophoresis and further cloned in pGEM^®^-T EASY vector (Promega, USA) and sequenced.

### 5’ and 3’ RACE (Rapid Amplification of cDNA Ends) of *ZoCDPK1*

5’ and 3’-RACE ready cDNA synthesis was performed with First Choice RLM RACE Kit (Ambion, USA) as per manufacturer’s protocol. For 5’ RACE, 10 μg of total RNA after CIP treatment, TAP treatment and adapter ligation was reverse transcribed using a random primer provided by the kit. 5’ RACE primary PCR was carried out in a 50 µL reaction mixture containing 1.0 µL of 5’ RACE ready cDNA, 5x Buffer with MgCl_2,_ 200 μM of each dNTPs, 20 pmol of 5’ RACE outer primer (5’-GCTGATGGCGATGAATGAACACTG-3’), 20 pmol of gene specific outer primer CD1AS1 (5’- ATACAGTATGAACAAGATAACCC-3’), and 3 U of *Taq* DNA polymerase (Promega, USA). The cDNA was denatured at 94°C for 3 min followed by 35 cycles (94°C for 30 sec, 60°C for 30 sec and 72°C for 2 min) and a final incubation at 72°C for 7 min. One μL of primary PCR product was used for secondary PCR using 5’ RACE inner primer (5’-CGCGGATCCGAACACTGCGTTTGCTGGCTTTGAT-3’) and gene specific inner primer CD1AS2 (5’-CATTGGAAGCCTCCTTGGCATTTT-3’) using the same above conditions. The nested PCR product was cloned into pGEMT-Easy vector and sequenced. For 3’ RACE, 1 μg of total RNA was reverse transcribed using 3’ RACE adapter provided by the kit. 3’ RACE primary PCR was carried out in a 50 µL reaction mixture containing 1.0 µL of 3’ RACE ready cDNA, 5x Buffer with MgCl_2,_ 200 μM of each dNTPs, 20 pmol of 3’ RACE outer primer (5’-GCGAGCACAGAATTAATACGACT-3’), 20 pmol of gene specific outer primer CD1S1 (5’-GGGGTTATCTTGTTCATACTGCTA-3’), and 3 U of *Taq* DNA polymerase (Promega, USA). The cDNA was denatured at 94°C for 3 min followed by 35 cycles (94°C for 30 sec, 60°C for 30 sec and 72°C for 2 min), and a final incubation at 72°C for 7 min. One μL of primary PCR product was used for secondary PCR using 3’ RACE inner primer (5’-CGCGGATCCGAATTAATACGACTCACTATAGG-3’) and a gene specific inner primer CD1S2 (5’-ATTCTCCGAGGGATGATTGATTT-3’) using the same above conditions. The nested PCR product was cloned into pGEMT-Easy vector and sequenced.

### Generation of full length cDNA of *ZoCDPK1* and domain analysis

Based on the nucleotide sequence of 5’ and 3’ RACE products, GSPF (5’-ATGGGAAATTCC TTCGTCTGCTGCG- 3’) and GSPR (5’-AGACTGTTATGACGGATGGTTTGCG-3’) were designed for the amplification of full length cDNA of *ZoCDPK1*. The PCR was carried out in a 50 µL reaction mixture containing 1.0 µL of cDNA (prepared after salinity induction), 10x Buffer with MgCl_2,_ 200 μM of each dNTPs, 50x Advantage 2 Polymerase mix (Clontech, Japan) and 20 pmol each of GSPF and GSPR. The cDNA was denatured at 95°C for 1 min followed by 30 cycles (95°C for 30 sec, 68°C for 3min) and a final incubation at 70°C for 10 min. The product was cloned into pGEMT-Easy vector and sequenced. The nucleotide and amino acid sequences of ZoCDPK1 were subjected to analysis using BLASTN, PSI-BLAST and PHI-BLAST. Sequences of other members of CDPKs were retrieved from GenBank (www.ncbi.nih.gov/). CDPK sequences containing NLS in their JD were identified manually analyzing all entries. Domain analysis was done using PROSITE (http://expasy.ch/prosite/) and SMART database (http://smart.embl-heidelberg.de/).

### Phylogenetic analysis

The phylogenetic tree was developed using Neighbour Joining (NJ) method implemented in the MEGA 4 software. All necessary alignments were performed with ClustalW [32]. The robustness of the tree topology was assessed by boot strap analysis with 1000 re sampling replicates.

### Quantitative real time PCR

To assay the expression of *ZoCDPK1* against various stresses, quantitative real-time PCR analysis was carried out on cDNAs prepared after various stress and phytohormone treatments. The endogenous control used to normalize variance in the quantity of RNA and the amount of cDNA was elongation factor 1 α (*EF1α*) gene, for which the conserved region was amplified from ginger. The gene *EF1α* is reported to be the most stable housekeeping gene during abiotic stress conditions [33]. Real-time PCR was performed on an optical 96-well plate with an ABI PRISM 7900HT Fast real-time PCR system (Applied Biosystems) using the primer pairs 5’-CCAAGGCCAAGTCCAAGGAA-3’ (forward primer) and 5’-TTCCC GGAGGACGTTGATG-3’ (reverse primer) for *ZoCDPK1* and 5’-GCTGACTGTGCTGTTCTC ATTATTG-3’ (forward primer) and 5’-CTCGTGTCTGTCCATCCTTTGAAA-3’ (reverse primer) for *EF1α*. Each reaction contained 12.5 μL of 2X Taqman Universal mastermix (Applied Biosystems, USA), 1.0 μL of cDNA samples and 1.0 μL of Taq assay mix in a final volume of 25.0 μL. The thermal cycle used was as follows: 50°C for 2 min, 95°C for 10 min followed by 40 cycles of 95°C for 15 sec and 60°C for 1 min. To study the expression of stress-inducible genes in transgenic tobacco plants quantitative real-time PCR studies were carried out in optical 96-well plate including 12.5 μL 2X SYBR Green Master mix reagent (Applied Biosystems, USA), 1.0 μL cDNA sample and 0.2 mM of each gene-specific primers in final volume of 25 μL using the thermal cycles as follows: 50°C for 2 min, 95°C for 10 min; followed by 40 cycles of 95°C for 15 sec and 60°C for 1 min. Dissociation curve analysis was performed as follows: 95°C for 15 sec; 60°C for 15 sec. The primers used were 5’-AGG ATGGCAAGGACTACTGGAT-3’ (*RD21A* forward primer), 5’-TGGATTGGCACCTACCTTTA TT-3’ (*RD21A* reverse primer); 5’-TTAGATGCCATAAATGCTGC-3’ (*ERD1* forward primer), 5’-TTGCCTGGACAGCCCTAATC-3’ (*ERD1* reverse primer); 5’-AGTGGAGCAACTGTGGGA CG-3’ (*AP2* forward primer) and 5’-TTCCTTTGAGCCCTTGTCTT-3’ (*AP2* reverse primer); 5’-ATCACTTGGCTCCACTGTTGTTC-3’ (*RD29A* forward primer), 5’-ACAAAACACACATAA ACATCCAAAGT-3’ (*RD29A* reverse primer); 5’-AAGAAAACAGGCGACAAGAT-3’ (*DREB1A* forward primer), 5’-ACGAAGCACAAAAAACTAGC-3’ (*DREB1A* reverse primer). Three independent experiments, each one in triplicates, were carried out in each case and relative gene expression was calculated by the equation 2^-ddCt^.

### Generation of ZoCDPK1*-*GFP fusion construct and development of transgenic tobacco lines

The binary vector pMDC85 [34] was used to prepare the *ZoCDPK1* expression construct for tobacco transformation. The full-length cDNA of *ZoCDPK1* was amplified using gene specific primers (GSPF and GSPR) and TA cloned into pCR^®^8/GW/TOPO entry vector (Invitrogen, USA) allowing the addition of attL1 and attL2 sites on the left and right of the insert. One entry clone was fully sequenced before subsequent cloning in the binary Gateway destination vector pMDC85 having compatible attR1 and attR2 sites. The *ZoCDPK1* was cloned in the sense orientation into pMDC85 vector using a Gateway LR clonase enzyme mix technology (Invitrogen, USA). The destination vector, pMDC85 allows the expression of the cDNA under the control of the dual 35S CaMV promoter and the C-terminus of ZoCDPK1 was fused to the N-terminus of GFP. The resulting binary vector, pMDC85-ZoCDPK1-GFP was introduced into the *Agrobacterium tumefaciens* strain EHA105, and used for transforming *Nicotiana tabaccum* var. petita hybrida leaf disks by the standard *Agrobacterium*-mediated transformation [35] method. The transformants were selected on hygromycin (25 mg/L). *Nicotiana tabacum* var. petita hybrid transformed with pMDC85 vector alone was used as vector control (VC) in all experiments. The seeds (T0 seeds) were collected and germinated on hygromycin-containing medium for raising transgenic T1 seedlings. Transgenic tobacco plants with hygromycin resistance were transferred to pots containing vermiculate and finally to earthen pots filled with soil, compost, and sand (1:1:1).

Five independent homozygous ZoCDPK1 over-expression transgenic lines (CD-1, CD-3, CD-5, CD-6 and CD-7) were developed. PCR analysis was carried out on T0 and T3 transgenic lines carrying ZoCDPK1. Genomic DNA was isolated from healthy leaves of four week old plants using GenElute Plant Genomic DNA isolation kit (Sigma, India) and amplification done using ginger *CDPK1* specific primers as described earlier. Total protein was isolated from T3 transgenic plants by TCA-acetone method [36] and twenty micrograms of protein were electrophoretically separated on 12.5% SDS-PAGE and transferred onto a nitrocellulose membrane. The membranes were blocked and thereafter blotted with a GFP specific monoclonal antibody (Sigma, India) for 3 h at a 1:5000 dilution. After extensive washing, the bound primary antibody was detected with a horseradish peroxidase-conjugated anti-mouse IgG secondary antibody using the 3, 3’- diaminobenzidine (DAB) assay.

### Transgenic plant material and stress treatments

To conduct stress treatments, wild type, vector control and T2 transgenic seeds (T3 generation) of two independent lines (CD-1 and CD-6) were surface sterilized with 70% ethanol and sown on 1/2 MS medium supplemented with 200 mM NaCl (salt stress) or 300 mM Mannitol (drought stress). For vector control and transgenic seeds, hygromycin was also included in the medium. The plates were placed in growth chamber (16 h light/8 h darkness, 24°C). The germination percentages were measured after 14 days of sowing. In order to monitor the effect of salinity stress, four week old T3 transgenic (CD-1 and CD-6), vector control and wild type plants were treated with 0 or 200 mM NaCl/300 mM mannitol every fourth day for four weeks. Growth characteristics like shoot length, root length, leaf area and plant dry weight were measured at four weeks after initiating the treatment. Shoot and root length was measured on meter scale. Leaf area was measured by a leaf area meter (Systronics, India). For physiological assessment, the wild-type, vector control and T3 transgenic plants (CD-1 and CD-6) were grown in growth chamber under 16 h light/8 h darkness at 24°C. These plants were then subjected to salinity and water deficit stresses, and four weeks after stress initiation, data were recorded for relative water content (RWC) and chlorophyll. The RWC were determined as described by Goel et al. [37] using the formula, RWC (%) = (FW-DW)/TW-DW) x 100. Chlorophyll content was measured spectrophotometrically after extraction in 80% acetone [38]. The tolerance indexes of salinity/drought stress treated plants were calculated using the data of plant dry weight by the following formula:

TI (%) = (plant dry weight with stress) / (plant dry weight with water) x 100

### Leaf disk assay

Leaf disk senescence assay were carried out on wild and transgenic plants (T3) to assess its stress tolerance as described by Tuteja [39] with some modifications. In brief, leaf disks of 1.0-cm diameter were excised from healthy and fully expanded tobacco leaves of similar age from transgenic, vector control and wild type (WT) plants (45 days old). The disks were floated in a 6-ml solution of NaCl or Mannitol or water (experimental control) for 96 h and then used for measuring chlorophyll a and b spectrophotometrically after extraction in 80% acetone [38]. The treatment was carried out in continuous white light at 25+2°C. The experiment was repeated at least three times with different transgenic lines.

### Determination of photosynthetic parameters

Gas exchange measurements were done on intact mature leaves of wild type, vector control and T3 transgenic plants (CD-1 and CD-6) kept at growth chamber using a Portable Photosynthesis System (LI-6400XT, Li-COR, U.S.A.). All the photosynthetic measurements were made at a leaf temperature of 30±2.0 °C and at a constant CO_2_ concentration of 400 µmol mol^-1^ using a CO_2_ injector (LI-6400-01, Li-COR, U.S.A.). For light response curve, the light intensity was varied from 20 to 1800 µmol quanta m^-2^ s^-1^ with CO_2_ assimilation values being logged for each light. Chlorophyll fluorescence measurements were made following standard technique as proposed by Schreiber et al. [40]. The maximum potential photochemical efficiency defined as the ratio of variable to maximum fluorescence emitted by chlorophyll (Fv/Fm) was estimated using a portable chlorophyll fluorometer PAM-2100 (Heinz Walz, Germany). The plants were dark adapted for 20 minutes prior to measurement. Maximal fluorescence under light exposure (Fm’) was obtained by imposing 1 sec saturating flash to the leaf in order to reduce the entire PS II reaction centre after attaining steady state fluorescence (Ft). Minimal fluorescence immediately after light exposure (Fo’) was determined by imposing dark while a far red light was simultaneously switched on to oxidize PS II rapidly by drawing electrons from PS II to PS I.

### Yield characteristics

At maturity wild type, vector control and T3 transgenic plants were harvested and the yield characteristics like time required for flowering, seed weight were scored as indicator for salinity and drought stress tolerance.

### Statistical analysis

Three independent experiments, each one in triplicates, were carried out in each case and representative data were shown. Student’s t-test was used to analyze all the data presented as the mean + SE (n=3) to compare the obtained parameters from Transgenic and wild type under normal or stress conditions. A P value of 0.0001 was considered to be statistically significant.

### Onion inner epidermal transformation and confocal laser-scanning microscopy (CLSM)

The protocol for onion inner epidermal peel preparation and Agrobacterium-mediated transformation were similar to those used for tobacco transformation as described by Wydro et al. [41] with some modifications. Agroinfiltration method of transient gene expression was carried out on healthy and fresh onion scales leaves under normal, salinity and drought stress conditions. The scales were rinsed with water, onion inner epidermal cell layers were peeled, adequately sliced, and stained with propidium iodide (PI) and imaged in water on a glass slide and covered with a cover slip. CLSM was performed using a Leica TCS NT/SP microscope with excitation/emission wavelengths 480/510 nm for GFP and 536/617 nm for PI.

## Results

### Isolation, cloning and sequencing of a CDPK from ginger

Total RNA was isolated from young leaves of ginger after salinity stress induction and reverse-transcribed to cDNA. A cDNA fragment of approximately 600 bp was amplified by RT-PCR using degenerate oligos based on conserved regions of other known CDPKs in the database. The full length cDNA was isolated through RLM-RACE approach with two sets of gene specific primers designed based on its core sequence. The full length cDNA, represented as *ZoCDPK1* and its accession number at GenBank was KC544003. The gene is 2074 bp long and contains an open reading frame of 1629 bp encoding 542 amino acids. It has a 135 bp 5’ UTR with an in-frame stop codon and a 300 bp 3’ UTR followed by a poly A tail, two characteristics indicative of a full-length sequence. The 1.6 kb ORF was amplified by PCR by using gene specific forward and reverse primers (GSPF and GSPR) from salinity stress induced cDNA.

**Figure 1:**
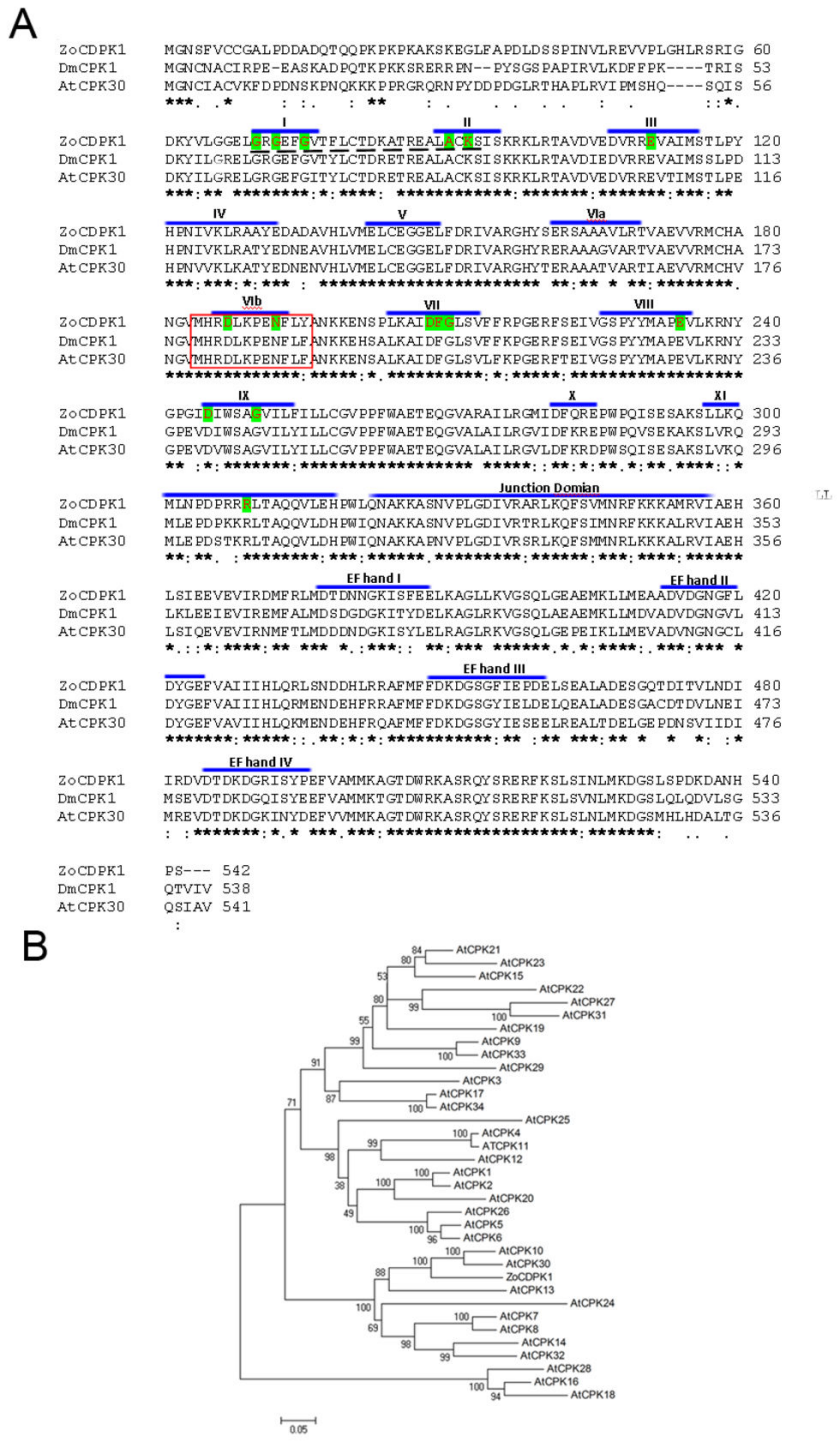
Amino acid sequence alignment and phylogenetic tree of ginger CDPK1. (A) The amino acid sequence alignment of *Zingiber* CDPK1 [GenBank ID: KC544003] with *Datura* CDPK, DmCPK1 [GenBank ID: 163658596] and *Arabidopsis* CDPK, AtCPK30 [GenBank ID: 30699042]. Identical amino acids are indicated by asterisks, and similar amino acids are marked with single dots and colons. Catalytic domains (I–XI), Junction domain, and EF hand loops of CaM-LD domain of ZoCDPK1 are marked. The 15 invariant amino acid residues for eukaryotic Ser/Thr protein kinase were highlighted. Protein kinase ATP-binding site is shown by broken lines. Active site is shown in red box. (B) Phylogenetic analysis of ZoCDPK1 with all *Arabidopsis* CDPKs in the database. The tree was constructed using NJ method of Mega4 software. The numbers on branches showed bootstrap probabilities determined for 1000 re-samplings. The database accession numbers are indicated in parantheses after CDPK gene names.

The deduced protein of ZoCDPK1 has 542 amino acids with a predicted molecular mass of 60.81 kD and isoelectric point of 6.77. Within its polypeptide ZoCDPK1 contains a long variable domain preceding a Ser/Thr protein kinase catalytic domain, an autoinhibitory function domain, a CaM-LD containing four EF hand Ca^2+^-binding motifs and a C-terminal variable domain as revealed by SMART and PROSITE analysis (Figure 1A). The N-terminal variable domain, kinase domain, JD, CaM-LD and C-terminal variable domains consist of 62, 259, 46, 140 and 35 amino acid residues, respectively. The kinase domain contains subdomains I to XI and 15 invariant amino acid residues for eukaryotic Ser/Thr protein kinase [42] including a possible ATP-binding site in the N-terminal region as shown in Figure 1A. ZoCDPK1 showed highest homology with DmCDPK1, a *Datura metel* CDPK (78% amino acid identity, GenBank accession number ABY28389). Other close homologues include PtCDPK10 (77% identity, XP002318616), from *Populus trichocarpa*; AtCPK30 (75% identity, NP177612), an *Arabidopsis thaliana* CDPK. The alignment of ZoCDPK1 with DmCDPK1 and AtCPK30 is shown in the Figure 1A.The phylogenetic tree based on ZoCDPK1 and all *Arabidopsis* CDPKs reported so far (Figure 1B) suggested that ZoCDPK1 was clustered along with AtCPK30, which is an osmotic stress responsive form.

**Figure 2:**
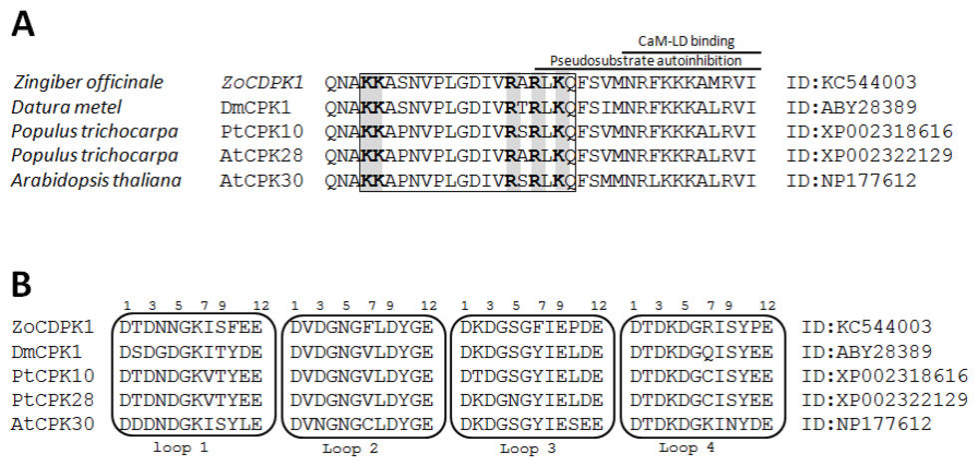
Domain analysis of ZoCDPK1. (A) Multiple sequence alignment of the JDs of CDPKs containing a bipartite NLS as a subdomain. The NLS is shown in the black box. The relative positions of autoinhibitory and CaM-LD binding sub-domains are indicated. The determinant amino acids for the bipartite NLS are shaded. (B) Multiple sequence alignment of Ca^2+^-binding EF hand loops of the above CDPKs [DX(DNS)(ILVFYW) (DENSTG)(DNQGHRK)(GP)(LIVMC)(DENQSTAGC)X(2)(DE)(LIVMFYW]. The residue numbers important for coordination with Ca^2+^ in the respective Ca^2+^-binding loops are indicated.

ZoCDPK1 with a typical core domain arrangement of a Ca^2+^ regulated kinase, also possess a bipartite nuclear localization sequence (NLS; PROSITE code PS50079) in its Junction domain. The sequence of all four Ca^2+^ binding EF hand loops (PROSITE code PS00018) in its CaM-LD are conserved (Figure 2). Further protein-protein and PSI-BLAST searches at NCBI database and subsequent domain analysis revealed that many other CDPKs from different species showing strong homology with ZoCDPK1 also have similar type of domain composition. As indicated in Figure 2, a novel coupling was noticed between the presence of a bipartite NLS in the junction domain with presence of consensus in the EF hand loops of CaM-LD. Multiple sequence alignment revealed that the position of the NLS in the JD of all these CDPKs were superimposed (Figure 2A). Canonical bipartite NLS contains (i) two adjacent basic amino acids (Arg or Lys), (ii) followed by spacer region of any 10 residues, and (iii) followed by at least three basic residues (Arg or Lys) in the next five positions [43]. ZoCDPK1 possess a typical Ca^2+^-binding EF hand loop with a consensus 12-residue (PROSITE code PS00018), which is flanked on both sides by a 12-residue α-helical domain. The calcium ion is co-ordinated in a pentagonal bipyramidal configuration with six residues in positions 1, 3, 5, 7, 9 and 12 (Figure 2B). The invariant Glu or Asp at position 12 provides two oxygen for liganding Ca^2+^ (bidentate ligand).

### Expression of *ZoCDPK1* in different parts of ginger

**Figure 3:**
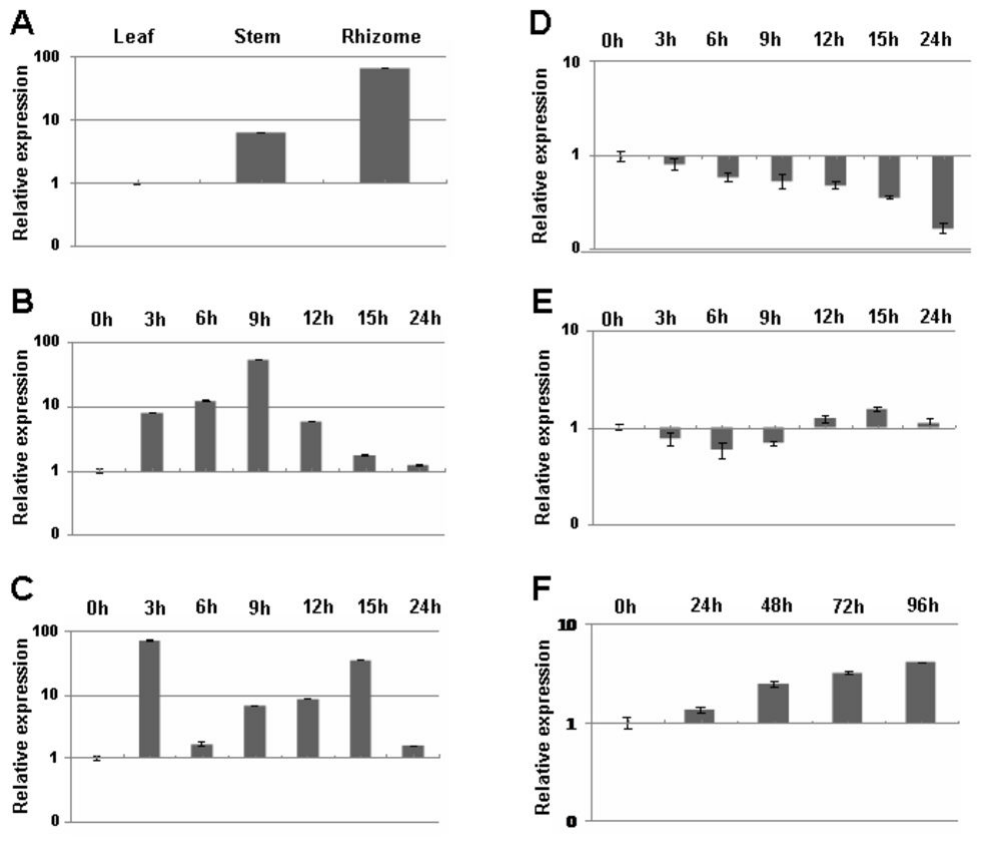
Quantitative real time PCR analysis of *ZoCDPK1* transcripts in ginger. (A) *ZoCDPK1* expression was studied in leaf, stem and rhizome. (B) Total RNA was isolated from leaves collected at successive intervals of 0, 3, 6, 9, 12, 15 and 24 h post salinity (400 mM NaCl). (C) Total RNA was isolated from leaves collected at successive intervals of 0, 3, 6, 9, 12, 15 and 24 h post drought (dehydration) treatment. (D) Total RNA was isolated from leaves collected at successive intervals of 0, 3, 6, 9, 12, 15 and 24 h post ABA (100 μM). (E) Total RNA was isolated from leaves collected at successive intervals of 0, 3, 6, 9, 12, 15 and 24 h post low temperature (4°C) treatment. (F) A time course expression profile of *ZoCDPK1* against JA (100 µM) was studied at regular intervals of 0, 24, 48, 72 and 96 h post treatment. The *EF1α* gene was used as endogenous control in all experiments and relative gene expression was calculated by the equation 2^-ddCt^. Data are presented as mean + SE (n=3) and error bars represent SE.

To examine the expression pattern of *ZoCDPK1* in leaves, stem and rhizome of ginger, quantitative real time PCR experiments were conducted on the cDNAs prepared from respective parts. The qRT-PCR data (Figure 3A) revealed that *ZoCDPK1* transcripts were present in all the tested plant parts including leaf, stem and rhizome with a very strong expression was noticed in rhizome.

### Expression of *ZoCDPK1* was induced by salt, drought and exogenous JA but not by ABA or cold treatments

To investigate whether *ZoCDPK1* expression is regulated by different stresses, treatments were given to 4-week-old ginger plants and leaf samples were collected as described in the Materials and Methods section. Total RNA was isolated, respective cDNAs were prepared and real-time PCR analysis was performed with *EF1α* gene as endogenous control. Time-course induction of *ZoCDPK1* expression by salinity and drought stresses were analyzed (Figure 3B and C). There was a basal level of *ZoCDPK1* expression at time 0 h, and the *ZoCDPK1* transcripts were increased at 3 h post stress treatment. It was found that *ZoCDPK1* transcripts accumulation reached its maximum at time point 9 h against salinity stress and decreased thereafter (Figure 3B). Against drought stress, maximum expression level of *ZoCDPK1* was noticed at 3 h itself (Figure 3C).

As many drought-inducible genes were shown to be responsive to exogenous ABA, we also examined the effect ABA on the expression of *ZoCDPK1*. Interestingly it was found that the expression of *ZoCDPK1* was progressively decreased by the exogenous application of 100 μM ABA (Figure 3D). The effect of low temperature (4°C) on the expression of *ZoCDPK1* was also tested. As shown in Figure 3E, *ZoCDPK1* was not responsive to low temperature. The marginal up-regulation during longer period as shown in the Figure 3E may be due to the overlapping physical stress. JA and methyl jasmonate (MeJA), collectively termed jasmonates, are regarded as endogenous regulators that play important roles in stress responses, plant growth and development [44]. It has been reported that jasmonate-responsive genes can also be induced by osmotic stress [45]. Hence the effect of JA on *ZoCDPK1* expression was also tested. As shown in the Figure 3F, *ZoCDPK1* expression increased marginally up to 24 h of JA treatment and rapidly thereafter. Based on all these expression data, we concluded that the expression of *ZoCDPK1* gene is induced by high salt, drought and JA rather than by cold or ABA signaling pathway in ginger plants.

### ZoCDPK1 over-expression and analysis of transgenic plants

**Figure 4:**
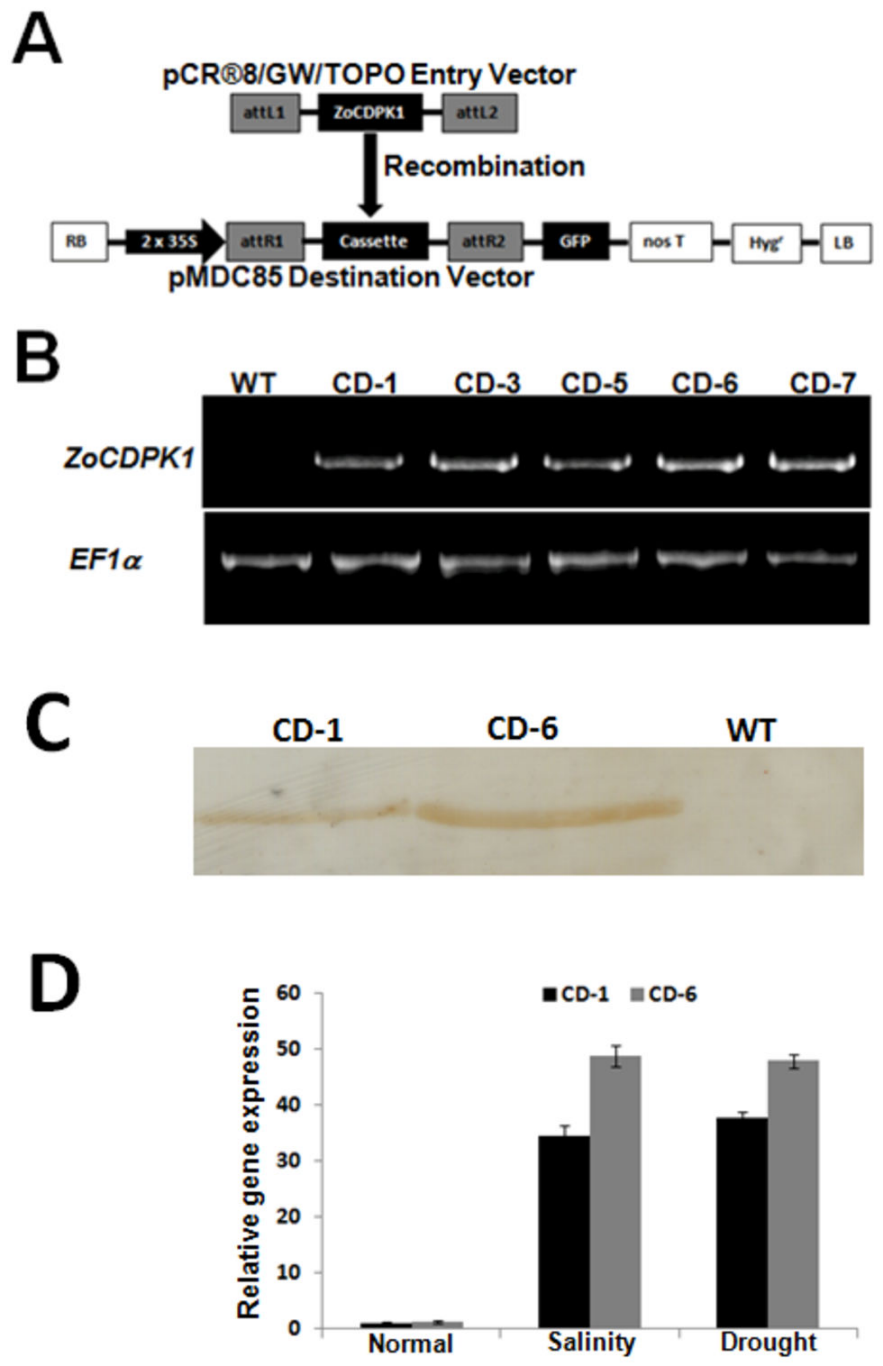
Schematic representation of the T-DNA and molecular analysis of *ZoCDPK1-*expressing tobacco. (A) Gateway recombination reaction between pCR^®^8/GW/TOPO-ZoCDPK1 entry vector and pMDC85 destination vector and resultant T-DNA region of the vector pMDC85-ZoCDPK1-GFP. RB, right T-DNA border; LB, left T-DNA border; 35S, cauliflower mosaic virus 35S promoter; nos T, NOS terminator; Hyg^r^, hygromycin phosphotransferase II gene. (B) RT-PCR analysis using *ZoCDPK1* specific primers and *EF1α* specific primers of wild type and five independent T3 transgenic lines. (C) Western blot analysis of wild type and and transgenic tobacco (CD-1 and CD-6) lines. Proteins (20 µg per lane) were fractionated by 12.5% SDS-PAGE and immunobloted with GFP-specific antibody (D) Quantitative real time PCR analysis of *ZoCDPK1* transcripts in wild type and transgenic tobacco lines (CD-1 and CD-6) under normal, salinity and drought conditions. The *EF1α* gene was used as endogenous control in all experiments. Relative gene expression was given as the fold expression change using the equation 2^-ddCt^. Data are presented as mean + SE (n=3) and error bars represent SE.

To evaluate *in vivo* physiological role of ZoCDPK1, the complete ORF was over-expressed in tobacco plants by using Agrobacterium-mediated transformation. For that the full length cDNA was placed under the control of 2X 35S promoter in pMDC85 gateway destination vector in such a manner that, intact GFP (green ﬂuorescent protein) coding sequence was fused at its C-terminus (Figure 4A). A total of 25 independent transgenic tobacco lines were generated, grew them to maturity; and five 5 homozygous transgenic lines over-expression *ZoCDPK1* were developed and confirmed by RT-PCR analysis (Figure 4B). Two dominant lines, CD-1 and CD-6 were selected and homozygous T3 plants were used for salinity and drought stress tolerant analysis. The transcription level of ZoCDPK1 was relatively high in CD-6 compared with that in CD-1 as revealed by RT-PCR analysis (Figure 4B). Similarly, the protein level of ZoCDPK1 was also relatively higher in CD-6 than in CD-1 when detected by Western blotting using a GFP-tag monoclonal antibody (Figure 4C). In *ZoCDPK1* over-expressing plants, the *ZoCDPK1* transcripts were accumulated at high concentration. The level was increased further under salinity and drought stress (Figure 4D). The general differences between wild type plants and plants transformed with empty vector were not significant for the studied parameters.

### Sub-cellular localization of ZoCDPK1

**Figure 5:**
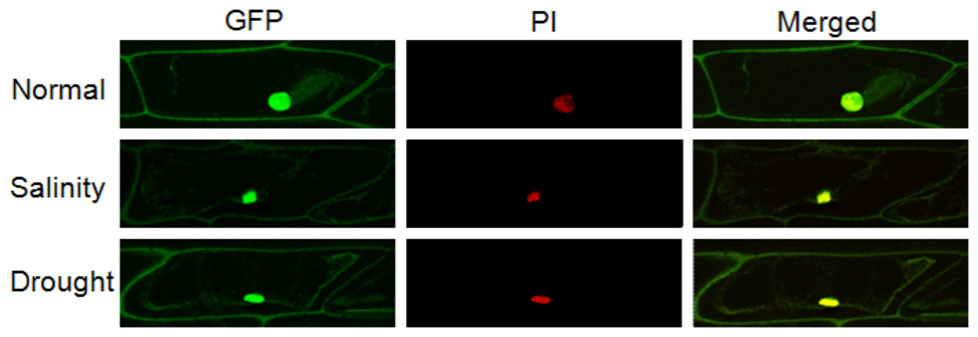
Sub-cellular localization of ZoCDPK1-GFP fusion protein. Transient expression of the ZoCDPK1-GFP in the plasmolyzed onion epidermal cells. Onion epidermal peels were incubated in IM sucrose for 10 minute before staining and visualization. GFP fluorescence, PI fluorescence and merged images are respectively the first, middle and last panels.

To investigate the sub-cellular localization of ZoCDPK1 during normal, salinity and drought stress, the ZoCDPK1-GFP construct was introduced into the onion epidermal cells by agroinfiltration method of transient transformation. As displayed in Figure 5, ZoCDPK1-GFP fusion protein was predominantly localized in the nucleus in all tested conditions. Under normal condition additional GFP fluorescence were also detected at a reasonable level in cytosol. Interestingly during salinity and drought stress conditions, additional signals were detected in plasma membrane and not in cytosol. To date this is the most common localization pattern for CDPK isoforms [14]. These results clearly indicate that the ZoCDPK1 protein possesses information to direct the nuclear targeting and moreover, the protein shows differences in localization pattern in response to stress conditions.

### Transgenic tobacco plants over-expressing *ZoCDPK1* show tolerance to excess salinity and drought stress

**Figure 6:**
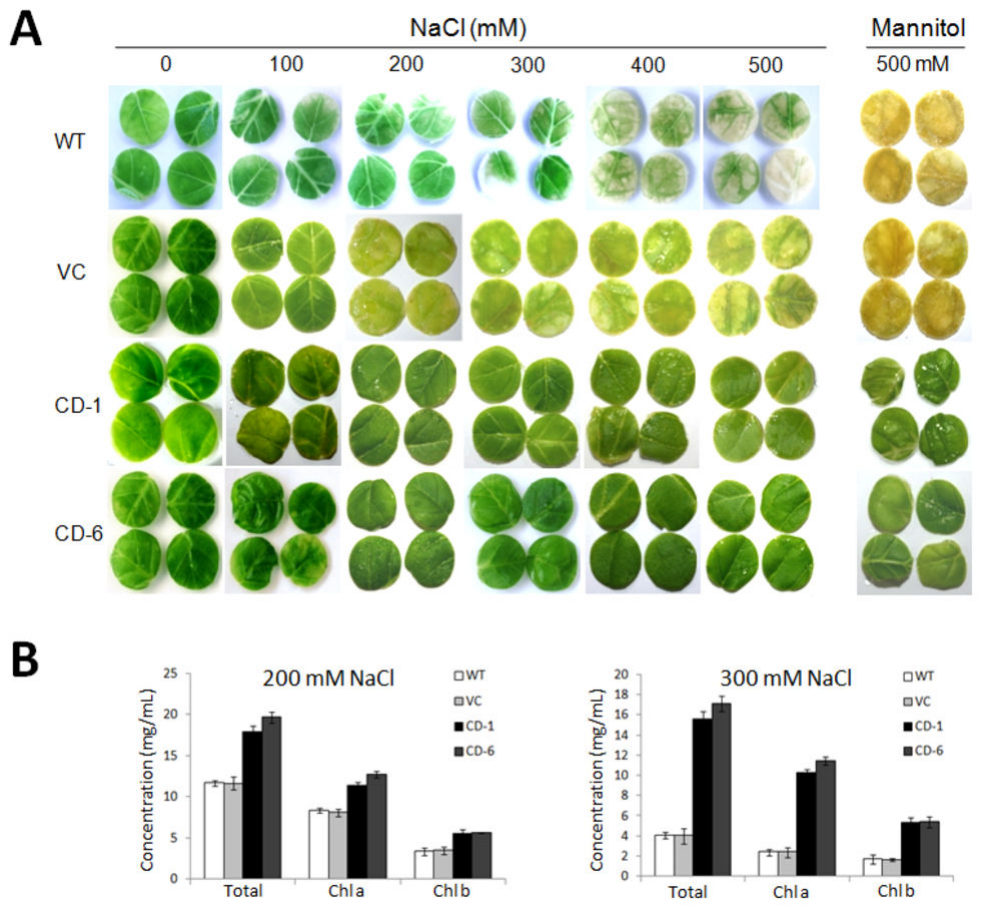
Leaf disk senescence assay for salinity and drought tolerance in transgenic tobacco plants (T3). (A) Representative pictures to show phenotypic differences in leaf disks of wild type (WT), vector control (VC) and T3 plants (CD-1 and CD-6) after incubation in 0, 100, 200, 300, 400 and 500 mM NaCl or 500 mM mannitol solutions. (B) Representative diagram to show the chlorophyll content from leaf disks of wild type, vector control and T3 transgenic plants after incubation in 200 mM NaCl or 300 mM mannitol solutions. Data are presented as mean + SE (n=3) and error bars represent SE. Data shows a significant difference of chl.a or chl.b content between transgenic and wild type controls at P<0.0001, by Student’s t-test.

To test for osmotic stress tolerance, leaf disks from CD-1 and CD-6 transgenic (T3), vector control and WT tobacco plants were floated separately on 100, 200, 300, 400 and 500 mM NaCl (salinity stress) or 300 mM mannitol (drought stress) and H_2_O for 96 h. Stress-induced loss of chlorophyll was lower in *ZoCDPK1* over-expressing lines compared with those from the WT plants (Figure 6A). The damage caused by stress was reflected in the degree of bleaching observed in the leaf tissue after 96 h. It was evident that transgenic plants have a better ability to tolerate salinity and drought stress. Measurement of chlorophyll content of the leaf disks from transgenic, vector control and wild type plants stressed with NaCl or mannitol (Figure 6B) provided further support for a positive relationship between the over-expression of *ZoCDPK1* and tolerance of osmotic stress.

**Figure 7:**
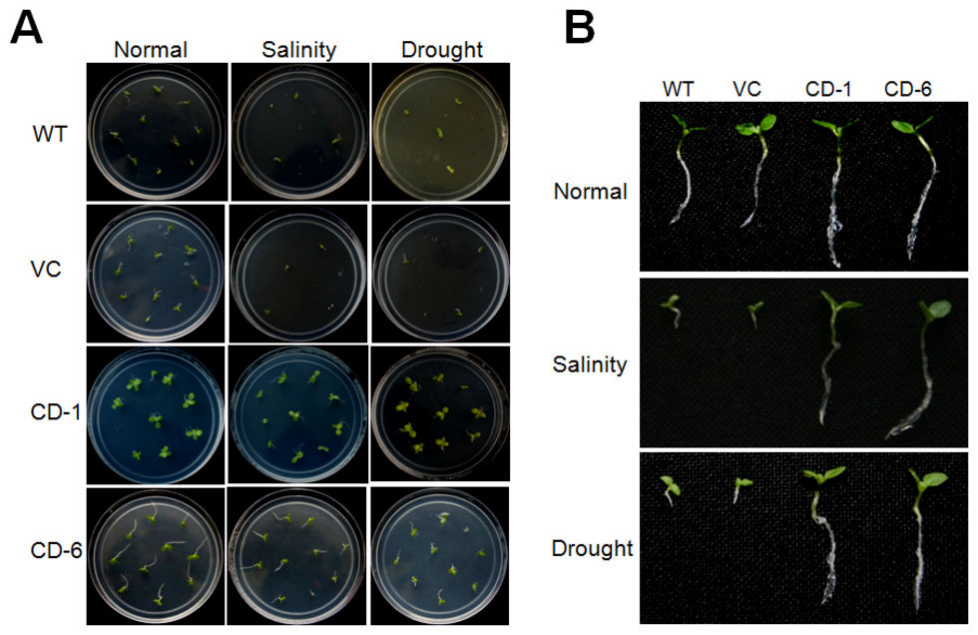
Analysis of ZoCDPK1 transgenic (T3) plants under salinity and drought. (A) Representative pictures to show the germination of transgenic (CD-1 and CD-6), vector control (VC) and wild type (WT) seeds under normal conditions (non-stress), 200 mM NaCl (salinity) and 300 mM mannitol (drought). (B) Representative seedlings of WT, VC and T3 homozygous lines (CD-1 and CD-6) taken after 14 days of germination on normal (non-stress), 200 mM NaCl (Upper) and 300 mM mannitol (Lower) conditions.

**Figure 8:**
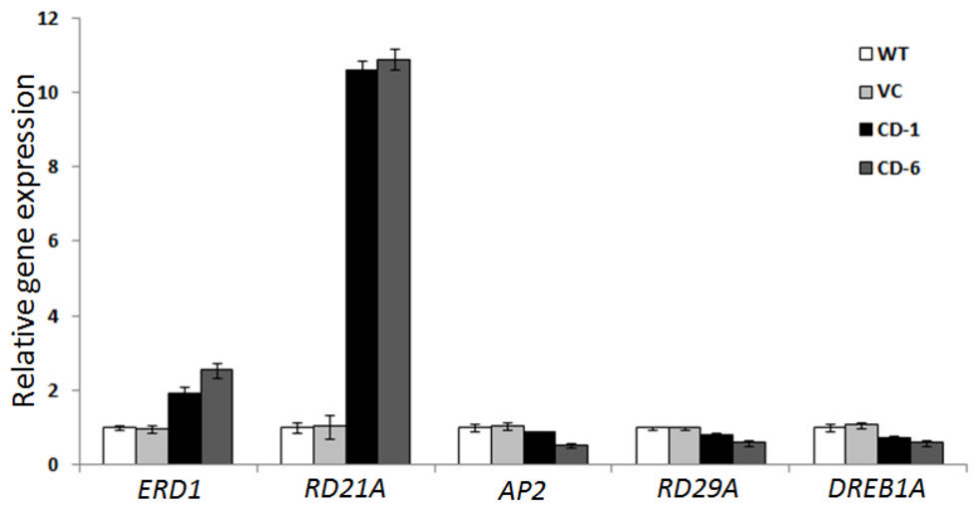
Quantitative real time PCR analysis of stress responsive genes in wild type, vector contorl and T3 transgenic plants after induction with 200 mM NaCl. Relative gene expression was given as the fold expression change using the equation 2^-ddCt^. Data are presented as mean + SE (n=3) and error bars represent SE.

Seeds obtained from T2 transgenic plants, vector control and wild type plants were germinated on 1/2 MS medium with 200 mM NaCl (salt stress) or 300 mM Mannitol (drought stress) stress. The transgenic seeds were able to germinate in the presence of 200 mM NaCl or 300 mM mannitol whereas the wild type and vector control seeds were highly susceptible to both the stress conditions and failed to germinate (Figure 7A and B). To assess the stress tolerance of intact *ZoCDPK1* over-expressing plants, the green house grown T3 lines (CD-1 and CD-6), vector control and wild type plants were watered with 200 mM NaCl solution. In the presence of NaCl, the wild type and vector control plants showed growth retardation, whereas transgenic plants did not develop any sign of stress (Figure S1). To elucidate further the role of ZoCDPK1 in stress tolerance, we examined the effects of *ZoCDPK1* over-expression on the transcript levels of different stress responsive genes – *ERD1* [46], *RD21A* [47], *AP2* [48], *RD29A* [49] and *DREB1A* [50] in leaves under salt stress. The stress responsive genes, *ERD1* and *RD21A* were up-regulated in the transgenic plant as compared to wild type and vector control plants during salinity stress whereas *AP2*, RD29A and DREB1A transcripts were slightly down-regulated (Figure 8).

### Physiological assessment of transgenic plants under normal, salinity and drought stress

Transformants of the T3 generation were used for the physiological analysis. T3 plants of both lines (CD-1 and CD-6) showed, on average, 73% more growth and contained 42% more chlorophyll per unit leaf area as compared to their wild type lines at normal conditions itself. A corresponding increase in shoot length, root length, leaf area, plant dry weight, chlorophyll a, chlorophyll b and total chlorophyll were observed between transgenic and wild type or vector control plants under non-stressed condition (Table 1). The differences were further pronounced when grown in presence of 200 mM NaCl or 300 mM mannitol. Salinity and drought stress induced reduction in growth parameters were given on Table 1. When the transgenic (T3) and wild-type plants were subjected to 200 mM NaCl, the relative water content (RWC) was 93% and 94% in CD-1 and CD-6 respectively, while it was only 74% in the wild-type plant. After four week of drought stress treatment, the RWC in the transgenic plants varied from 90-91% whereas it was 69% in the wild-type plants. Tolerance index of T3 transgenics and wild type plants against salinity/drought has also been calculated using the data of plant dry weight at 0 or 200 mM NaCl/300 mM mannitol. Interestingly, the tolerance potential of T3 transgenic plants were 81.8% to 82.6%, whereas, it was just 31.8% to 35.7% in the case of wild type plants. The yield parameters like time required for flowering, seed weight/pod are found to be better for the T3 transgenic lines at 200 mM NaCl or 300 mM mannitol than wild type plants grown without stress treatments (Table 2). The growth chamber grown CD-6 transgenic line (T3) also showed an early flowering when grown on salinity stress in comparison to wild type plant, which showed growth retardation and early symptoms of withering (Figure S2).

**Table 1:** Comparison of shoot length (SL, cm/plant), root length (RL, cm/plant), leaf area (LA, cm^2^/plant), plant dry weight (DW, g/plant), chlorophyll a (Chl a, mg/g fresh weight), chlorophyll b (Chl b, mg/g fresh weight) and total chlorophyll (Total Chl, mg/g fresh weight) of WT, VC and T3 transgenic plants (CD-1 and CD-6) grown under normal (non-stress), salinity (200 mM NaCl) and drought (300 mM Mannitol) conditions.

**Clone**	**Condition**	**Parameters**						
		SL	RL	LA	DW	Chl a	Chl b	Total Chl
**WT**	Normal	138±1.9	18.3±1.0	132±8.9	22.21±1.3	5.92±0.21	2.42±0.16	8.34±0.3
	Salinity	33±0.8^c^	6.6±0.7^c^	37±1.6^c^	7.06±0.9^c^	3.36±0.10^c^	1.44±0.14^c^	4.80±0.2^c^
	Drought	27±1.4^c^	6.4±0.4^c^	38±6.8^c^	7.92±1.2^c^	2.32±0.13^c^	1.01±0.16^c^	3.33±0.4^c^
**VC**	Normal	137±1.7	18.2±1.0	130±7.9	23.10±1.1	5.79±0.33	2.44±0.26	8.24±0.3
	Salinity	33±0.6^c^	6.7±0.8^c^	35±1.6^c^	7.04±1.1^c^	3.38±0.30^c^	1.37±0.14^c^	4.75±0.3^c^
	Drought	26±1.7^c^	6.3±0.5^c^	37±8.6^c^	7.93±1.0^c^	2.39±0.23^c^	1.00±0.13^c^	3.39±0.4^c^
**CD-1**	Normal	238±10.3^a^	29.3±1.3^a^	230±7.9^a^	37.62±1.3^a^	7.93±0.26^a^	3.82±0.16^a^	11.75±0.3^a^
	Salinity	210±12.6^b^	26.4±0.8^b^	202±9.8^b^	30.84±1.7^b^	7.20±0.22^b^	2.75±0.12^b^	9.95±0.5^a^
	Drought	207±9.3^b^	26.1±0.9^b^	201±7.4^b^	31.03±1.4^b^	7.50±0.21^b^	3.24±0.21^a^	10.74±0.4^a^
**CD-6**	Normal	240±10.9^a^	31.3±1.2^a^	234±7.8^a^	38.74±1.5^a^	8.41±0.27^a^	3.48±0.30^a^	11.89±0.5^a^
	Salinity	211±13.1^b^	27.0±1.0^b^	203±7.9^b^	31.72±1.7^b^	7.40±0.27^b^	2.73±0.27^b^	10.13±0.3^a^
	Drought	209±10.4^b^	27.1±1.3^b^	202±10.2^b^	32.01±1.6^b^	7.11±0.22^b^	2.78±0.17^b^	9.89±0.5^a^

Each value represents mean of three replicates ± SE (n = 3). Data followed by the same letter in the same column are not significantly different at P<0.05 as determined by Duncan’s multiple range tests.

**Table 2:** Comparison of flowering time and seed weight of wild type and T3 transgenic plants (CD-1 and CD-6) grown under normal (non-stress), salinity (200 mM NaCl) and drought (300 mM Mannitol) conditions.

**Parameter**	**Water grown**	**200 mM NaCl**		**300 mM mannitol**	
	Wild type#	CD-1	CD-6	CD-1	CD-6
**Flowering time (Days)**	128±1.45	107±1.08	109±1.53	107±0.93	108±1.21
Seed weight/pod* (Milligrams)	156±1.91	162±2.80	164±2.63	164±2.10	163±2.70

Each value represents mean of three replicates ± SE (n = 3). # Wild type plants did not survived under salinity/drought stress. * The data recorded from plants harvested at maturity (~150 days).

**Figure 9:**
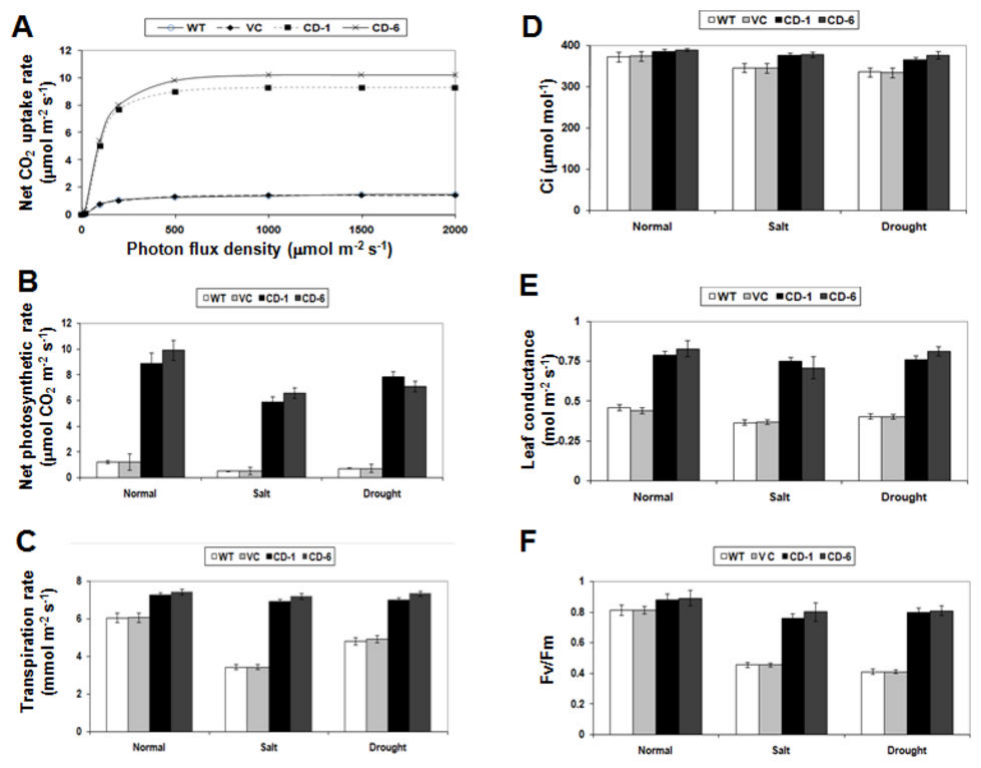
Physiological assessment of transgenic plants (T3), vector control and wild type plants. (A) Net CO_2_ uptake rate measured at a PPFD of 20 and 2000 µmol quanta m^-2^ sec^-1^ and 400 p.p.m. CO_2_. Effect of salinity and drought stress on the net photosynthetic rate (B), Transpiration rate (C), inter-cellular CO_2_ concentration (D), leaf conductance (E) and photosynthetic efficiency (F) of transgenic and wild type plants.

The physiological observations summarized above suggested that ZoCDPK1 over-expression resulted in improved plant growth under normal condition itself, which further enhanced upon stress treatments. Therefore, ZoCDPK1 over-expression may have caused a concerted stimulation of cellular and physiological processes that regulate plant vigor. To test this speculation, we examined several representative physiological parameters that control plant vigor: (1) light response curve or net CO_2_ uptake rate, which reveals characteristics of the underlying photosynthesis processes including the light-dependent and light-independent reactions, the efficiency at which light is utilized by photosynthesis, and even the rate of O_2_ uptake; (2) photosynthesis rate, a trait positively correlated with plant vitality and biomass production; (3) transpiration rate, a trait generally associated with the rates of water consumption and transport in the plant; (4) intercellular CO_2_ concentration (Ci) and leaf conductance, are the measures of the rate of passage of carbon dioxide (CO_2_) entering, or water vapor exiting through the stomata of a leaf; and (5) photochemical quantum efficiency (measured as the chlorophyll fluorescence parameter Fv/Fm [maximum photochemical efficiency of photosystem II in the dark-adapted state]), a trait positively correlated with the organization and vitality of photosystem II. The transgenic plants over-expressing ZoCDPK1 had significantly higher net CO_2_ uptake rate, photosynthesis rates, transpiration rates, leaf conductance, Ci and Fv/Fm than the wild type or vector control plants (Figure 9A, B, C, D, E and F respectively) under normal conditions, which upon stress elicitation manifold to a great extent. These results suggest that under salinity and drought stress conditions, the transgenic plants had much better growth than the control plants at normal/stress conditions.

## Discussion

CDPKs have been reported to function in response to cytoplasmic Ca^2+^ elevations in many physiological processes in plants [4]. Considering the status of ginger as one of the important and value spice, an understanding of the calcium signaling associated with abiotic stimuli at the molecular level would undoubtedly have enormous benefits. Although many studies have shown the involvement of Ca^2+^-dependent protein kinases in abiotic stress signal transduction and plant stress tolerance [3], a CDPK with biological bipartite NLS, and is having conserved EF-hand residues, that function as positive transducer in these responses have remained unknown. In this study the cloning and characterization of multiple-stress inducible CDPK gene from ginger (*ZoCDPK1*) with a functional bipartite NLS is discussed in relation to salinity and drought stress.

### Domain analysis of ZoCDPK1; novel coupling between nuclear localization sequences in the JD and consensus EF-hand loops in the CaM-LD

The sequence homologies of ginger CDPK, ZoCDPK1 with different plant CDPKs suggest that the isolated gene encodes a CDPK. ZoCDPK1 also possess four consensus Ca^2+^-binding sites as would expected for a functional canonical CDPK (Figure 1). ZoCDPK1 shows high homology with AtCPK30, a salt and drought stress inducible CDPK from *Arabidopsis* [51]; AtCPK10, an *Arabidopsis* CDPK involved in dehydration, high salt and ABA signaling [22,52], and CaCDPK4, a salinity stress, JA responsive CDPK from *Capsicum annuum* [53]. In ZoCDPK1 a novel coupling between the occurrence of an NLS in the JD with conserved residues in EF hand loop were noticed. This is a deviation from the earlier report which discusses that for the functional exposition of NLS in JDs, some sort of compensatory change in the total kinase like non-functional EF hand loops required [54]. Such CDPKs are activated only after binding with protein of importin family and hence functions in the nucleus only. The presence of four conserved EF-hands in CaM-LD domain of ZoCDPK1, nullified the need of importin binding for its activation. Hence a detailed database search was conducted and we could find some other CDPKs with similar type of coupling and they also show high homology with ZoCDPK1 (Figure 2). But the exact reason for this type of coupling remains to be elusive. Because the characteristic calcium-dependent structural response in the CaM-LDs is reported to be associated with the regulatory apparatus of canonical CDPKs [55] the principle of activation of the *Zingiber* CDPK appears to remain unchanged. Altogether it may be presumed that ZoCDPK1 is a functional CDPK with typical Ca^2+^-binding properties and extra bipartite NLS in its JD and hence have activity in the nucleus.

### Differential expression of *ZoCDPK1* gene in ginger

Some CDPK genes are expressed in all plant organs or tissues, but their mRNAs are especially abundant in given organs or tissues. The mRNA and protein of VfCPK1, a stress inducible calcium-dependent protein kinase from *Vicia faba*, were expressed in all organ or tissues tested, with its abundance in epidermal peels and leaves [56]. Similarly CaCPK1 transcripts and protein from chick pea accumulated in all the organ samples examined with maximum expression of transcripts in roots [57]. In the present study, mRNA of *ZoCDPK1* was detected in all tissues examined; however its expression was more abundant in rhizome than in leaf or stem. The exact significance of this observation is yet to be determined, but it suggests a differential regulation of metabolism, Ca^2+^ signaling and development between these tissue types. This observation was supported by the recent findings from the EST database of ginger, that many signal transduction genes were expressed at higher levels in rhizomes than in leaves [58]. Several such genes that are up-regulated in the rhizome or expressed exclusively in the rhizome are candidate genes that may be involved in determining cell fate and tissue differentiation leading to rhizome development and maintenance [58]. Raíces et al. [59] reported a temporal correlation between an increase in CDPK (StCDPK1) activity and the morphological changes associated with the onset of tuber (modified underground stem like rhizome) development using *in vitro* cultured potato stolons. *StCDPK1* expression was shown to be specifically up-regulated by sucrose and sorbitol; hence osmotic stress activation of sucrose-phosphate synthase leading to *in vitro* tuber induction was discussed. In continuum StCDPK2, a potato CDPK isoform, phosphorylates StABF1, a tuber development responsive bZIP transcription factor *in vitro* [60].

### Expression of *ZoCDPK1* was induced by osmotic stress and JA but not by low temperature and ABA treatments

*CDPK* genes were shown to be differentially regulated. Either dehydration or exposure to high concentrations of NaCl induced both *AtCPK10* and *AtCPK11* but not by ABA [22]. *VfCPK1*, a CDPK gene from broad bean, is transcriptionally regulated by drought, ABA and CaCl_2_ [56]. Over-expression of rice *OsCDPK7* yielded cold and salt/drought tolerant rice plants [26] while another rice CDPK, OsCPK12 acts as a positive regulator of salt tolerance [61]. *OsCDPK13* expression was increased in leaf sheath segments of rice treated with gibberellin or subjected to cold stress [62]. More over tobacco CDPKs, NtCDPK2 and NtCDPK3 were shown to be differentially phosphorylated *in vivo* as part of a kinase cascade that regulates abiotic stress response [63]. These results suggest that *CDPK* genes are differentially induced at the transcription level by various stimuli involved in the abiotic stress signal cascades.

The ginger CDPK, *ZoCDPK1* gene is transcriptionally up-regulated by salt stress, drought and JA but not by ABA or low temperature. Upon drought and salt stress, the level of *ZoCDPK1* transcripts started to increase immediately after stress treatment, became highly induced at 3 h and 9 h respectively and went back to the basal level at 24 h post-treatment. As there is no up-regulation of *ZoCDPK1* transcripts by ABA even up to 24 h, it may be possible that ABA is not involved in the induction of *ZoCDPK1* by dehydration. In *Arabidopsis* endogenous ABA-induced by water stress deficiency is detectable by 2 h after dehydration and reaches a maximum level by 10 h [64], providing further evidence that ABA is not involved in the induction of *ZoCDPK1*. Against JA, a progressive increase in expression of CDPK was noticed in *Solanum tuberosum* [27]. Overall these results show that a change in osmotic potential triggers the rapid induction of *ZoCDPK1*.

Many abiotic-stress-inducible genes are controlled by ABA, but some are not, which indicates that both ABA-dependent and ABA-independent regulatory systems are involved in stress-responsive gene expression [65-67]. The salinity stress-induced upregulation of transcript of *PDH45* was reported to follow an ABA-dependent pathway [68], while MCM6 from pea followed the ABA-independent pathway [69]. More than that, the *Arabidopsis* Ca^2+^-dependent protein kinase CPK12 acts as a negatively regulator in ABA signal transduction [70]. DRE/CRT/LTRE is a major *cis*-acting regulatory element in ABA-independent gene expression under abiotic stress conditions [71] and is also found in the promoter region of genes which are induced by both drought and cold [65,72]. We found that *ZoCDPK1* is induced by drought and salinity but not by ABA or low temperature (Figure 3B, C, D and E), which suggests that DRE is not likely to function in their rapid dehydration-responsive expression.

### Ginger CDPK1 has a dynamic localization pattern

In plants CDPKs exhibit multiple locations, including the cytosol, nucleus, plasma membrane, peroxisomes [14], endoplasmic reticulum [15], mitochondria [73] and oil bodies [74]. Similarly many CDPKs with potential mono or bipartite NLS showed nuclear localization under normal or stressed conditions. In the case of tomato CDPK, LeCPK1, the nuclear targeting may result from a potential NLS located close to the C-terminal of kinase domain [75] whereas in AtCPK11, the calcium-dependent protein kinase from *Arabidopsis*, the nuclear localization is effected by having two potential NLS [76]. Interestingly, a bipartite NLS is present in its junction domain for the groundnut CDPK, AhCPK2, and the protein showed nuclear localization too [54]. Due to the absence of N-terminal myristoylation sequence and the presence of NLS in JD, it was presumed that ZoCDPK1 may exist as a soluble form with possible nuclear localization. The sub-cellular localization of ZoCDPK1 was investigated under normal and stress conditions with confocal microscopy in onion epidermal peel cells. The CLSM images of ZoCDPK1-GFP fusion proteins confirmed its localization in nucleus and cytosol at normal conditions. At drought and salinity stress conditions, the fusion protein localize in nucleus and plasma membrane. Patharkar and Cushman [77] showed that McCDPK1, a membrane bound CDPK from ice plant (*Mesembryanthemum crystallinum*) localized in the nucleus during salt or dehydration stress and also phosphorylated CDPK substrate protein 1 (CSP1), which is a transcription factor belonging to a class of two-component pseudo-response regulators. In contrast ZoCDPK1 shows nuclear localization under normal and stressed conditions. In response to stress ZoCDPK1 additionally localizes to plasma membrane rather than cytosol, which is the additional location during normal condition.

### Over-expression of *ZoCDPK1* changed expression of ABA-independent genes

The functional analysis of the role of ZoCDPK1 in stress tolerance was studied by over-expressing *ZoCDPK1* in tobacco. It was found that the fresh weight, retention of green color, as well as percentage of seed germination were much better in transgenic lines, as compared with vector control or wild type tobacco, under normal or stress conditions. The induction of numerous stress responsive genes is a hallmark of stress adaptation in plants [78]. Here we observed that constitutive expression of *ZoCDPK1* under the control of 35S promoter triggered an altered expression of various stress responsive genes such as *ERD1*, *RD21A*, *AP2*, *RD29A* and *DREB1A* in tobacco. In a similar study, Xu et al. [23] has reported that over-expression of *AtCPK6* activated the expression level of *RD29A*, *RD29B* and *RD22*.

There are several drought-inducible genes that do not respond to either cold or ABA treatment, suggesting the existence of other ABA-independent pathway in the dehydration stress response [71]. ABA-independent stress-responsive gene expression is regulated by DREB proteins that bind to DRE/CRT *cis*-elements [79]. Transcription factors belonging to AP2 family and DREB1A specifically bind to the DRE/CRT sequence and activate the transcription of cold-inducible genes [50,80]. ERD1 (EARLY RESPONSIVE TO DEHYDRATION 1), which encodes a Clp protease regulatory subunit; ClpD is reported to be up-regulated in response to drought, high salinity but not with cold or ABA treatment [81]. The *RD29A* gene expression is induced by high salinity, dehydration, low temperature [49]. In addition to this, several drought and high salinity stress-inducible genes such as *RD19* and *RD21* that encode different cysteine proteases [47] are induced through the ABA-independent pathway [81]. Neither of their mRNA synthesis was responsive to cold stress. The over-expression of *ZoCDPK1* in tobacco up-regulated *ERD1* and *RD21A* transcripts whereas *AP2*, *RD29A* and *DREB1A* transcripts were almost unaffected. This result confirms the earlier observation from gene expression studies that, DRE/CRT is not likely to function in the dehydration response of ZoCDPK1 under salinity/drought stress.

### *ZoCDPK1* over-expressing plants shows increased growth and photosynthesis and set viable seeds without yield penalty under normal, salinity stress and drought stress

The ZoCDPK1 over-expressing T3 plants showed increased amounts of shoot length, root length, fresh weight, dry weight, net chlorophyll content, relative water content, and relative humidity in comparison to vector control and wild type plants under normal condition, salinity and drought stress. Moreover the T3 transgenic plants showed early flowering (17% reduction in flowering time) with almost equal seed weight under salinity/drought stress in comparison with wild types grown without stress. Since it is well known that photosynthesis and the electron transport system are adversely affected under salinity and drought stress [82], to assess the ability of the transgenic plants to ameliorate salt and drought effects on photosynthesis, various physiological parameters are measured. Significant difference were observed in the net CO_2_ uptake rate, photosynthetic rate, C*i*, leaf conductance, chlorophyll fluorescence parameter Fv/Fm and transpiration rate between transgenic, vector control and wild type plants. These results indicate a substantial protection of photosystem especially PSII during salinity and drought stress in transgenic plants. Hence the ZoCDPK1 over-expressing plants have greater adaptability and efficiency to capture maximum available light towards driving more photosynthesis. Since the T3 seedlings were able to grow, flower, and sets viable seeds under high salinity stress, the introduced trait is stable and functional in transgenic plants. However, the exact mechanism of *ZoCDPK1* mediated tolerance of salinity and drought stress is not understood. Based on the properties of ZoCDPK1, we suggest there may be two site of action of this kinase: 1) ZoCDPK1 might interacts with some transcription factors of NAC mediated ABA-independent pathway in the nucleus which directly involved in stress adaptation, 2) ZoCDPK1 might interacts with enzymes associated with photosynthetic regulation which resulted in enhanced growth and yield.

## Conclusions

Our finding revealed that *ZoCDPK1* gene from ginger could be a positive regulator involved in the adaptation of ginger against salt and drought and is a valuable candidate gene for crop improvement in the area of abiotic stress tolerance. Moreover salinity and drought tolerance of ZoCDPK1 over-expression plants likely through non-DRE/CRT mediated ABA-independent pathway. The interesting coupling between presence of NLS in the JD and conserved residues in the EF-hands of CDPK noticed in this study will provide an excellent start point to investigate further the role of NLS in the activation of CDPKs. The predominant occurrence of ZoCDPK1 in nucleus during stress/normal conditions justifies the requirement of a functional NLS in ZoCDPK1. Overall, this study will contribute to our better understanding of CDPK signaling and abiotic stress regulation in higher plants.

## Supporting Information

Figure S1 **Salinity stress tolerant phenotype of wild type, vector control and T3 transgenic plants grown in green house.** Wild type (WT), vector control (VC) and T3 tobacco (lines CD-1 and CD-6) in soil pots supplied with 200 mM NaCl solution and picture taken after six weeks of stress elicitation.
(TIF)

Figure S2 **Early flowering of growth chamber grown CD-6 transgenic plant (T3) in comparison to wild type under salinity stress.** Wild type and CD-6 tobacco line were in soil pots supplied with 200 mM NaCl solution.
(TIF)

## References

[1] Mahajan S, Tuteja N (2005) Cold, salinity and drought stresses: an overview. Arch Biochem Biophys 444: 139-158. doi:10.1016/j.abb.2005.10.018. PubMed: 16309626. 16309626

[2] Mahajan S, Pandey GK, Tuteja N (2008) Calcium- and salt-stress signaling in plants: shedding light on SOS pathway. Arch Biochem Biophys 471: 146-158. doi:10.1016/j.abb.2008.01.010. PubMed: 18241665. 18241665

[3] Kudla J, Batistic O, Hashimoto K (2010) Calcium signals: the lead currency of plant information processing. Plant Cell 22: 541-563. doi:10.1105/tpc.109.072686. PubMed: 20354197. 20354197 PMC2861448

[4] Harmon AC, Gribskov M, Harper JF (2000) CDPKs - a kinase for every Ca^2+^ signal? Trends Plant Sci 5: 154-159. doi:10.1016/S1360-1385(00)01577-6. PubMed: 10740296. 10740296

[5] Tuteja N, Mahajan S (2007) Calcium signaling network in plants: an overview. Plant Signal Behav 2: 79-85. doi:10.4161/psb.2.2.4176. PubMed: 19516972. 19516972 PMC2633903

[6] Harmon AC (2003) Calcium-regulated protein kinases of plants. Gravit Space Biol Bull 16: 83-90. PubMed: 12959135. 12959135

[7] Harper JF, Harmon AC (2005) Plants, symbiosis and parasites: a calcium signalling connection. Nat Rev Mol Cell Biol 6: 555–566. doi:10.1038/nrm1679. PubMed: 16072038. 16072038

[8] Christodoulou J, Malmendal A, Harper JF, Chazin WJ (2004) Evidence for differing roles for each lobe of the calmodulin-like domain in a calcium-dependent protein kinase. J Biol Chem 279: 29092-29100. doi:10.1074/jbc.M401297200. PubMed: 15126505. 15126505

[9] Asano T, Tanaka N, Yang G, Hayashi N, Komatsu S (2005) Genome-wide identification of the rice calcium-dependent protein kinase and its closely related kinase gene families: comprehensive analysis of the CDPKs gene family in rice. Plant Cell Physiol 46: 356-366. doi:10.1093/pcp/pci035. PubMed: 15695435. 15695435

[10] Cheng SH, Willmann MR, Chen HC, Sheen J (2002) Calcium signaling through protein kinases. The *Arabidopsis* calcium-dependent protein kinase gene family. Plant Physiol 129: 469-485. doi:10.1104/pp.005645. PubMed: 12068094. 12068094 PMC1540234

[11] Hrabak EM, Chan CW, Gribskov M, Harper JF, Choi JH et al. (2003) The *Arabidopsis* CDPK-SnRK superfamily of protein kinases. Plant Physiol 132: 666-680. doi:10.1104/pp.102.011999. PubMed: 12805596. 12805596 PMC167006

[12] Ray S, Agarwal P, Arora R, Kapoor S, Tyagi AK (2007) Expression analysis of calcium-dependent protein kinase gene family during reproductive development and abiotic stress conditions in rice (*Oryza* sativa L. ssp. indica). Mol Genet Genomics 278: 493-505. 17636330 10.1007/s00438-007-0267-4

[13] Harper JF, Breton G, Harmon A (2004) Decoding Ca^(2+)^ signals through plant protein kinases. Annu Rev Plant Biol 55: 263-288. doi:10.1146/annurev.arplant.55.031903.141627. PubMed: 15377221. 15377221

[14] Dammann C, Ichida A, Hong B, Romanowsky SM, Hrabak EM et al. (2003) Subcellular targeting of nine calcium-dependent protein kinase isoforms from *Arabidopsis* . Plant Physiol 132: 1840-1848. doi:10.1104/pp.103.020008. PubMed: 12913141. 12913141 PMC181270

[15] Lu SX, Hrabak EM (2002) An *Arabidopsis* calcium-dependent protein kinase is associated with the endoplasmic reticulum. Plant Physiol 128: 1008-1021. doi:10.1104/pp.010770. PubMed: 11891256. 11891256 PMC152213

[16] Asano T, Hayashi N, Kikuchi S, Ohsugi R (2012) CDPK-mediated abiotic stress signaling. Plant Signal Behav 7: 817-821. doi:10.4161/psb.20351. PubMed: 22751324. 22751324 PMC3583972

[17] Hubbard KE, Siegel RS, Valerio G, Brandt B, Schroeder JI (2012) Abscisic acid and CO_2_ signalling via calcium sensitivity priming in guard cells, new CDPK mutant phenotypes and a method for improved resolution of stomatal stimulus-response analyses. Ann Bot 109: 5-17. doi:10.1093/aob/mcr252. PubMed: 21994053. 21994053 PMC3241576

[18] Jaworski K, Pawełek A, Kopcewicz J, Szmidt-Jaworska A (2012) The calcium-dependent protein kinase (PnCDPK1) is involved in *Pharbitis* *nil* flowering. J Plant Physiol 169: 1578-1585. doi:10.1016/j.jplph.2012.05.025. PubMed: 22840323. 22840323

[19] Ma SY, Wu WH (2007) AtCPK23 functions in *Arabidopsis* responses to drought and salt stresses. Plant Mol Biol 65: 511-518. doi:10.1007/s11103-007-9187-2. PubMed: 17541706. 17541706

[20] Wan B, Lin Y, Mou T (2007) Expression of rice Ca^(2+)^-dependent protein kinases (CDPKs) genes under different environmental stresses. FEBS Lett 581: 1179-1189. doi:10.1016/j.febslet.2007.02.030. PubMed: 17336300. 17336300

[21] Yoon GM, Cho HS, Ha HJ, Liu JR, Lee HS (1999) Characterization of NtCDPK1, a calcium-dependent protein kinase gene in *Nicotiana* *tabacum*, and the activity of its encoded protein. Plant Mol Biol 39: 991-1001. doi:10.1023/A:1006170512542. PubMed: 10344204. 10344204

[22] Urao T, Katagiri T, Mizoguchi T, Yamaguchi-Shinozaki K, Hayashida N et al. (1994) Two genes that encode Ca^(2+)^-dependent protein kinases are induced by drought and high-salt stresses in *Arabidopsis* *thaliana* . Mol Gen Genet 244: 331-340. PubMed: 8078458. 8078458 10.1007/BF00286684

[23] Xu J, Tian YS, Peng RH, Xiong AS, Zhu B et al. (2010) AtCPK6, a functionally redundant and positive regulator involved in salt/drought stress tolerance in *Arabidopsis* . Planta 231: 1251-1260. doi:10.1007/s00425-010-1122-0. PubMed: 20217124. 20217124

[24] Mehlmer N, Wurzinger B, Stael S, Hofmann-Rodrigues D, Csaszar E et al. (2010) The Ca^(2+)^-dependent protein kinase CPK3 is required for MAPK-independent salt-stress acclimation in *Arabidopsis* . Plant J. PubMed: 20497378 10.1111/j.1365-313X.2010.04257.x PMC2988408 20497378

[25] Komatsu S, Yang G, Khan M, Onodera H, Toki S et al. (2007) Over-expression of calcium-dependent protein kinase 13 and calreticulin interacting protein 1 confers cold tolerance on rice plants. Mol Genet Genomics 277: 713-723. doi:10.1007/s00438-007-0220-6. PubMed: 17318583. 17318583

[26] Saijo Y, Hata S, Kyozuka J, Shimamoto K, Izui K (2000) Over-expression of a single Ca^2+^-dependent protein kinase confers both cold and salt/drought tolerance on rice plants. Plant J 23: 319-327. doi:10.1046/j.1365-313x.2000.00787.x. PubMed: 10929125. 10929125

[27] Ulloa RM, Raíces M, MacIntosh GC, Maldonado S, Téllez-Iñón MT (2002) Jasmonic acid affects plant morphology and calcium-dependent protein kinase expression and activity in *Solanum* *tuberosum* . Physiol Plant 115: 417-427. doi:10.1034/j.1399-3054.2002.1150312.x. PubMed: 12081535. 12081535

[28] Yu XC, Li MJ, Gao GF, Feng HZ, Geng XQ et al. (2006) Abscisic acid stimulates a calcium-dependent protein kinase in grape berry. Plant Physiol 140: 558-579. doi:10.1104/pp.105.074971. PubMed: 16407437. 16407437 PMC1361324

[29] Zhu SY, Yu XC, Wang XJ, Zhao R, Li Y et al. (2007) Two calcium-dependent protein kinases, CPK4 and CPK11, regulate abscisic acid signal transduction in *Arabidopsis* . Plant Cell 19: 3019-3036. doi:10.1105/tpc.107.050666. PubMed: 17921317. 17921317 PMC2174700

[30] Szczegielniak J, Borkiewicz L, Szurmak B, Lewandowska-Gnatowska E, Statkiewicz M et al. (2012) Maize calcium-dependent protein kinase (ZmCPK11): local and systemic response to wounding, regulation by touch and components of jasmonate signaling. Physiol Plant 146: 1-14. doi:10.1111/j.1399-3054.2012.01587.x. PubMed: 22289134. 22289134

[31] Wurzinger B, Mair A, Pfister B, Teige M (2011) Cross-talk of calcium-dependent protein kinase and MAP kinase signaling. Plant Signal Behav 6: 8-12. doi:10.4161/psb.6.1.14012. PubMed: 21248475. 21248475 PMC3121996

[32] Thompson JD, Higgins DG, Gibson TJ (1994) CLUSTAL W: improving the sensitivity of progressive multiple sequence alignment through sequence weighting, position-specific gap penalties and weight matrix choice. Nucleic Acids Res 22: 4673-4680. doi:10.1093/nar/22.22.4673. PubMed: 7984417. 7984417 PMC308517

[33] Nicot N, Hausman JF, Hoffmann L, Evers D (2005) Housekeeping gene selection for real-time RT-PCR normalization in potato during biotic and abiotic stress. J Exp Bot 56: 2907-2914. doi:10.1093/jxb/eri285. PubMed: 16188960. 16188960

[34] Curtis MD, Grossniklaus U (2003) A gateway cloning vector set for high-throughput functional analysis of genes in planta. Plant Physiol 133: 462-469. doi:10.1104/pp.103.027979. PubMed: 14555774. 14555774 PMC523872

[35] Horsch RB, Fry JE, Hoffmann NL, Eichholtz D, Rogers SG et al. (1985) A simple and general method for transferring genes into plants. Science 227: 1229-1231. doi:10.1126/science.227.4691.1229. PubMed: 17757866. 17757866

[36] Méchin V, Damerval C, Zivy M (2007) Total protein extraction with TCA-acetone. In: Plant proteomics: methods and protocols, vol 355, Methods in molecular biology. Clifton: Humana Press pp 1–8. 10.1385/1-59745-227-0:1 17093296

[37] Goel D, Singh AK, Yadav V, Babbar SB, Bansal KC (2010) Overexpression of osmotin gene confers tolerance to salt and drought stresses in transgenic tomato (*Solanum* *lycopersicum* L.). Protoplasma 245: 133-141. 20467880 10.1007/s00709-010-0158-0

[38] Lichtenthaler HK (1987) Chlorophylls and Carotenoids: Pigments of Photosynthetic Biomembranes. Methods Enzymol 148: 350-366. doi:10.1016/0076-6879(87)48036-1.

[39] Tuteja N (2010) A method to confer salinity stress tolerance to plants by helicase overexpression. Methods Mol Biol 587: 377-387. PubMed: 20225163. 20225163 10.1007/978-1-60327-355-8_26

[40] Schreiber U, Klughammer C, Neubauer C (1988) Measuring P700 absorbance changes around 830 nm with a new type of pulse modulation system. Z Naturforsch 43: 686-669.

[41] Wydro M, Kozubek E, Lehmann P (2006) Optimization of transient *Agrobacterium*-mediated gene expression system in leaves of *Nicotiana* *benthamiana* . Acta Biochim Pol 53: 289-298. PubMed: 16582986. 16582986

[42] Hanks SK, Quinn AM, Hunter T (1988) The protein kinase family: conserved features and deduced phylogeny of the catalytic domains. Science 241: 42-52. doi:10.1126/science.3291115. PubMed: 3291115. 3291115

[43] Dingwall C, Laskey RA (1991) Nuclear targeting sequences--a consensus? Trends Biochem Sci 16: 478-481. doi:10.1016/0968-0004(91)90184-W. PubMed: 1664152. 1664152

[44] Wasternack C (2007) Jasmonates: an update on biosynthesis, signal transduction and action in plant stress response, growth and development. Ann Bot 100: 681-697. doi:10.1093/aob/mcm079. PubMed: 17513307. 17513307 PMC2749622

[45] Schenk PM, Kazan K, Wilson I, Anderson JP, Richmond T et al. (2000) Coordinated plant defense responses in *Arabidopsis* revealed by microarray analysis. Proc Natl Acad Sci U S A 97: 11655-11660. doi:10.1073/pnas.97.21.11655. PubMed: 11027363. 11027363 PMC17256

[46] Kiyosue T, Yamaguchi-Shinozaki K, Shinozaki K (1993) Characterization of cDNA for a dehydration-inducible gene that encodes a CLP A, B-like protein in *Arabidopsis* *thaliana* L. Biochem Biophys Res Commun 196: 1214-1220. doi:10.1006/bbrc.1993.2381. PubMed: 7504470. 7504470

[47] Koizumi M, Yamaguchi-Shinozaki K, Tsuji H, Shinozaki K (1993) Structure and expression of two genes that encode distinct drought-inducible cysteine proteinases in *Arabidopsis* *thaliana* . Gene 129: 175-182. doi:10.1016/0378-1119(93)90266-6. PubMed: 8325504. 8325504

[48] Gilmour SJ, Zarka DG, Stockinger EJ, Salazar MP, Houghton JM et al. (1998) Low temperature regulation of the *Arabidopsis* CBF family of AP2 transcriptional activators as an early step in cold-induced COR gene expression. Plant J 16: 433-442. doi:10.1046/j.1365-313x.1998.00310.x. PubMed: 9881163. 9881163

[49] Narusaka Y, Nakashima K, Shinwari ZK, Sakuma Y, Furihata T, Abe H, Narusaka M, Shinozaki K, Yamaguchi-Shinozaki K (2003) Interaction between two *cis*-acting elements, ABRE and DRE, in ABA-independent expression of *Arabidopsis* *rd29A* gene in response to dehydration and high-salinity stresses. Plant J 34: 137-148. doi:10.1046/j.1365-313X.2003.01708.x. PubMed: 12694590. 12694590

[50] Kasuga M, Miura S, Shinozaki K, Yamaguchi-Shinozaki K (2004) A Combination of the Arabidopsis DREB1A Gene and Stress-Inducible rd29A Promoter Improved Drought- and Low-Temperature Stress Tolerance in Tobacco by Gene Transfer. Plant Cell Physiol 45: 346-350. doi:10.1093/pcp/pch037. PubMed: 15047884. 15047884

[51] Sheen J (1996) Ca^2+^-dependent protein kinases and stress signal transduction in plants. Science 274: 1900-1902. doi:10.1126/science.274.5294.1900. PubMed: 8943201. 8943201

[52] Zou JJ, Wei FJ, Wang C, Wu JJ, Ratnasekera D et al. (2010) *Arabidopsis* calcium-dependent protein kinase CPK10 functions in abscisic acid- and Ca^2+^-mediated stomatal regulation in response to drought stress. Plant Physiol 154: 1232-1243. doi:10.1104/pp.110.157545. PubMed: 20805328. 20805328 PMC2971602

[53] Chung E, Oh SK, Park JM, Choi D (2007) Expression and promoter analyses of pepper CaCDPK4 (*Capsicum* *annuum* calcium dependent protein kinase 4) during plant defense response to incompatible pathogen. Plant Pathol 23: 76-89. doi:10.5423/PPJ.2007.23.2.076.

[54] Raichaudhuri A, Bhattacharyya R, Chaudhuri S, Chakrabarti P, Dasgupta M (2006) Domain analysis of a groundnut calcium-dependent protein kinase: nuclear localization sequence in the junction domain is coupled with nonconsensus calcium binding domains. J Biol Chem 281: 10399-10409. doi:10.1074/jbc.M511001200. PubMed: 16464867. 16464867

[55] Vitart V, Christodoulou J, Huang JF, Chazin WJ, Harper JF (2000) Intramolecular activation of a Ca^(2+)^-dependent protein kinase is disrupted by insertions in the tether that connects the calmodulin-like domain to the kinase. Biochemistry 39: 4004-4011. doi:10.1021/bi992373m. PubMed: 10747788. 10747788

[56] Liu G, Chen J, Wang X (2006) VfCPK1, a gene encoding calcium-dependent protein kinase from Vicia faba, is induced by drought and abscisic acid. Plant Cell Environ 29: 2091-2099. doi:10.1111/j.1365-3040.2006.01582.x. PubMed: 17081243. 17081243

[57] Syam Prakash SR, Jayabaskaran C (2006) Expression and localization of calcium-dependent protein kinase isoforms in chickpea. J Plant Physiol 163: 1135-1149. doi:10.1016/j.jplph.2006.04.002. PubMed: 16716453. 16716453

[58] Gang DR, Ma X (2008) Ginger and Turmeric, Ancient Spices and Modern Medicines. In: Moore PH Ming R Genomics of Tropical Crop Plants. Springer Verlag.

[59] Raíces M, Gargantini PR, Chinchilla D, Crespi M, Téllez-Iñón MT et al. (2003) Regulation of CDPK isoforms during tuber development. Plant Mol Biol 52: 1011-1024. doi:10.1023/A:1025478315648. PubMed: 14558661. 14558661

[60] Muñiz García MN, Giammaria V, Grandellis C, Téllez-Iñón MT, Ulloa RM et al. (2012) Characterization of StABF1, a stress-responsive bZIP transcription factor from *Solanum* *tuberosum* L. that is phosphorylated by StCDPK2 in vitro. Planta 235: 761-778. doi:10.1007/s00425-011-1540-7. PubMed: 22042328. 22042328

[61] Asano T, Hayashi N, Kobayashi M, Aoki N, Miyao A et al. (2012) A rice calcium-dependent protein kinase OsCPK12 oppositely modulates salt-stress tolerance and blast disease resistance. Plant J 69: 26-36. doi:10.1111/j.1365-313X.2011.04766.x. PubMed: 21883553. 21883553

[62] Abbasi F, Onodera H, Toki S, Tanaka H, Komatsu S (2004) OsCDPK13, a calcium-dependent protein kinase gene from rice, is induced by cold and gibberellin in rice leaf sheath. Plant Mol Biol 55: 541-552. doi:10.1007/s11103-004-1178-y. PubMed: 15604699. 15604699

[63] Witte CP, Keinath N, Dubiella U, Demoulière R, Seal A et al. (2010) Tobacco calcium-dependent protein kinases are differentially phosphorylated in vivo as part of a kinase cascade that regulates stress response. J Biol Chem 285: 9740-9748. doi:10.1074/jbc.M109.052126. PubMed: 20118232. 20118232 PMC2843223

[64] Kiyosue T, Yamaguchi-Shinozaki K, Shinozaki K (1994) Cloning of cDNAs for genes that are early-responsive to dehydration stress (ERDs) in *Arabidopsis* *thaliana* L.: identification of three ERDs as HSP cognate genes. Plant Mol Biol 25: 791-798. doi:10.1007/BF00028874. PubMed: 8075396. 8075396

[65] Thomashow MF (1999) PLANT COLD ACCLIMATION: Freezing Tolerance Genes and Regulatory Mechanisms. Annu Rev Plant Physiol Plant Mol Biol 50: 571-599. doi:10.1146/annurev.arplant.50.1.571. PubMed: 15012220. 15012220

[66] Zhu JK (2002) Salt and drought stress signal transduction in plants. Annu Rev Plant Biol 53: 247-273. doi:10.1146/annurev.arplant.53.091401.143329. PubMed: 12221975. 12221975 PMC3128348

[67] Shinozaki K, Yamaguchi-Shinozaki K, Seki M (2003) Regulatory network of gene expression in the drought and cold stress responses. Curr Opin Plant Biol 6: 410-417. doi:10.1016/S1369-5266(03)00092-X. PubMed: 12972040. 12972040

[68] Sanan-Mishra N, Pham XH, Sopory SK, Tuteja N (2005) Pea DNA helicase 45 overexpression in tobacco confers high salinity tolerance without affecting yield. Proc Natl Acad Sci U S A 102: 509-514. doi:10.1073/pnas.0406485102. PubMed: 15630095. 15630095 PMC544286

[69] Dang HQ, Tran NQ, Gill SS, Tuteja R, Tuteja N (2011) A single subunit MCM6 from pea promotes salinity stress tolerance without affecting yield. Plant Mol Biol 76: 19-34. doi:10.1007/s11103-011-9758-0. PubMed: 21365356. 21365356

[70] Zhao R, Sun HL, Mei C, Wang XJ, Yan L et al. (2011) The *Arabidopsis* Ca^(2+)^ -dependent protein kinase CPK12 negatively regulates abscisic acid signaling in seed germination and post-germination growth. New Phytol 192: 61-73. doi:10.1111/j.1469-8137.2011.03793.x. PubMed: 21692804. 21692804

[71] Yamaguchi-Shinozaki K, Shinozaki K (2005) Organization of cis-acting regulatory elements in osmotic- and cold-stress-responsive promoters. Trends Plant Sci 10: 88-94. doi:10.5363/tits.10.10_88. PubMed: 15708346. 15708346

[72] Shinozaki K, Yamaguchi-Shinozaki K (2000) Molecular responses to dehydration and low temperature: differences and cross-talk between two stress signaling pathways. Curr Opin Plant Biol 3: 217-223. doi:10.1016/S1369-5266(00)00067-4. PubMed: 10837265. 10837265

[73] Pical C, Fredlund KM, Petit PX, Sommarin M, Møller IM (1993) The outer membrane of plant mitochondria contains a calcium-dependent protein kinase and multiple phosphoproteins. FEBS Lett 336: 347-351. doi:10.1016/0014-5793(93)80835-I. PubMed: 8262260. 8262260

[74] Anil VS, Harmon AC, Rao KS (2000) Spatio-temporal accumulation and activity of calcium-dependent protein kinases during embryogenesis, seed development, and germination in sandalwood. Plant Physiol 122: 1035-1043. doi:10.1104/pp.122.4.1035. PubMed: 10759499. 10759499 PMC58938

[75] Rutschmann F, Stalder U, Piotrowski M, Oecking C, Schaller A (2002) LeCPK1, a calcium-dependent protein kinase from tomato. Plasma membrane targeting and biochemical characterization. Plant Physiol 129: 156-168. doi:10.1104/pp.000869. PubMed: 12011347. 12011347 PMC155880

[76] Rodriguez Milla MA, Uno Y, Chang IF, Townsend J, Maher EA et al. (2006) A novel yeast two-hybrid approach to identify CDPK substrates: characterization of the interaction between AtCPK11 and AtDi19, a nuclear zinc finger protein. FEBS Lett 580: 904-911. doi:10.1016/j.febslet.2006.01.013. PubMed: 16438971. 16438971

[77] Patharkar OR, Cushman JC (2006) A novel coiled-coil protein co-localizes and interacts with a calcium-dependent protein kinase in the common ice plant during low-humidity stress. Planta 225: 57-73. doi:10.1007/s00425-006-0330-0. PubMed: 16773372. 16773372

[78] Pareek A, Sopory SK, Bohnert HJ (2010) Abiotic Stress Adaptation in Plants: Physiological, Molecular and Genomic Foundation. Springer Verlag.

[79] Agarwal PK, Agarwal P, Reddy MK, Sopory SK (2006) Role of DREB transcription factors in abiotic and biotic stress tolerance in plants. Plant Cell Rep 25: 1263-1274. doi:10.1007/s00299-006-0204-8. PubMed: 16858552. 16858552

[80] Shinozaki K, Yamaguchi-Shinozaki K (2007) Gene networks involved in drought stress response and tolerance. J Exp Bot 58: 221-227. PubMed: 17075077. 17075077 10.1093/jxb/erl164

[81] Nakashima K, Kiyosue T, Yamaguchi-Shinozaki K, Shinozaki K (1997) A nuclear gene, erd1, encoding a chloroplast-targeted Clp protease regulatory subunit homolog is not only induced by water stress but also developmentally up-regulated during senescence in *Arabidopsis* *thaliana* . Plant J 12: 851-861. doi:10.1046/j.1365-313X.1997.12040851.x. PubMed: 9375397. 9375397

[82] Silva EN, Ferreira-Silva SL, Fontenele Ade V, Ribeiro RV, Viégas RA et al. (2010) Photosynthetic changes and protective mechanisms against oxidative damage subjected to isolated and combined drought and heat stresses in Jatropha curcas plants. J Plant Physiol 167: 1157-1164. doi:10.1016/j.jplph.2010.03.005. PubMed: 20417989. 20417989

