# Supplementary figures and images for: CDPK1 from Ginger Promotes Salinity and Drought Stress Tolerance without Yield Penalty by Improving Growth and Photosynthesis in Nicotiana tabacum
Source: PLoS One. 2013 Oct 23;8(10):e76392. doi: 10.1371/journal.pone.0076392 (PMC3806807; doi:10.1371/journal.pone.0076392)

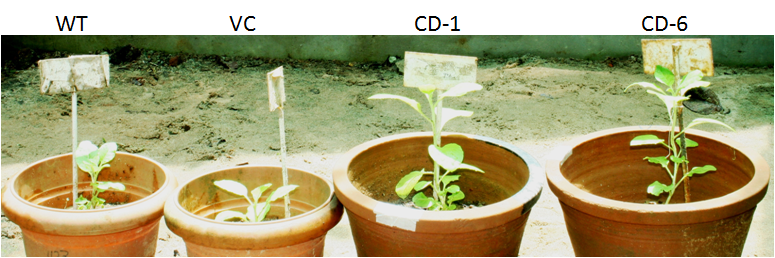

Supplement: Figure S1 — Salinity stress tolerant phenotype of wild type, vector control and T3 transgenic plants grown in green house. Wild type (WT), vector control (VC) and T3 tobacco (lines CD-1 and CD-6) in soil pots supplied with 200 mM NaCl solution and picture taken after six weeks of stress elicitation. (TIF) [file pone.0076392.s001.tif]

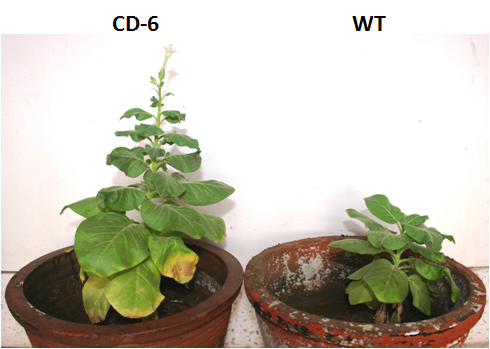

Supplement: Figure S2 — Early flowering of growth chamber grown CD-6 transgenic plant (T3) in comparison to wild type under salinity stress. Wild type and CD-6 tobacco line were in soil pots supplied with 200 mM NaCl solution. (TIF) [file pone.0076392.s002.tif]
